# Circ_0001741 exerts as a tumor promoter in ovarian cancer through the regulation of miR-491-5p/PRSS8 axis

**DOI:** 10.1007/s12672-024-01474-3

**Published:** 2024-11-11

**Authors:** Ding Wang, Sumin Zhang, Qiaoling Wang, Pengrong Li, Yunxia Liu

**Affiliations:** Department of Gynaecology, Yi Chang Maternal and Child Health Hospital, 99 Chengdong Avenue, Wujiagang District, Yichang City, 443001 Hubei China

**Keywords:** Ovarian cancer, Circ_0001741, MiR-491-5p, PRSS8

## Abstract

**Background:**

Circular RNAs (circRNAs) are important regulators for ovarian cancer (OC). Circ_0001741 has been found to be highly expressed in OC samples and is involved in regulating paclitaxel resistance in OC cells. Therefore, circ_0001741 may play a vital role in OC process, and its potential molecular mechanism is worth further revealing.

**Methods:**

Circ_0001741, miR-491-5p, and PRSS8 levels in OC tumor tissues and cells were quantified by quantitative real-time PCR or western blot. The proliferation, apoptosis and metastasis of OC cells were detected by cell counting kit 8 assay, Edu assay, flow cytometry, and transwell assay. RNA interaction was verified by dual-luciferase reporter assay and RIP assay. Xenograft assay was used to detect the effect of circ_0001741 knockdown on OC tumor growth in vivo.

**Results:**

Circ_0001741 was upregulated in OC tissues and cell lines. Knockdown of circ_0001741 repressed OC cell proliferation, metastasis, and enhanced apoptosis. Mechanistically, miR-491-5p was targeted by circ_0001741, and miR-491-5p inhibitor could attenuate the effect of circ_0001741 silencing on OC cell progression. Meanwhile, PRSS8 was a target of miR-491-5p, and miR-491-5p overexpression inhibited OC cell progression by targeting PRSS8. Circ_0001741 regulated PRSS8 expression by sponging miR-491-5p. Besides, circ_0001741 knockdown also inhibited OC tumor growth in vivo.

**Conclusion:**

Our data showed that circ_0001741 could promote the growth and metastasis of OC cells through the miR-491-5p/PRSS8 axis, which provided a potential molecular target for the treatment of OC.

**Supplementary Information:**

The online version contains supplementary material available at 10.1007/s12672-024-01474-3.

## Introduction

Ovarian cancer (OC) is the most common pernicious tumor in women, and the death rate is the supreme in gynecological vicious tumors [1]. OC is characterized by insidious early symptoms and lack of specific clinical manifestations, with a short survival time and a prognosis prone to recurrence [2]. The mortality rate of OC has fallen by over 30% in recent decades due to the reduced incidence and improved treatment [3]. However, the therapeutic effect of OC is still unsatisfactory. Therefore, the exploration of potential therapeutic targets for OC remains an important clinical issue that needs to be addressed urgently.

Circular RNA (circRNA) is mainly composed of pre-mRNA splicing and is a novel non-coding RNA that does not contain 5' end caps and 3' end poly **A** tails [4]. CircRNA participates in regulating the progression of many pernicious cancers, such as thyroid carcinoma [5], endometrial cancer [6], hepatocellular carcinoma [7]. CircRNA regulates tumor progression mainly by mediating cell biological process, including proliferation, metastasis, and apoptosis [8, 9]. Currently, a variety of circRNAs in OC are also aberrantly expressed. For example, circ_0061140 knockdown had been confirmed to repress OC cell proliferation and metastasis [10]. Besides, silencing of circ_0007841 restrained OC cell proliferation and invasion [11], and inhibition of circ_0005124 inhibited OC multiplication and metastasis [12]. However, the functions of many circRNAs haves not been fully investigated in OC.

Circ_0001741 is situated on chromosome 7, has a genomic length of 3179 bp and is derived from the back-cutting of TNPO3 mRNA (from exon 2 to exon 4). Previous study showed that circ_0001741 could upregulate NEK2 protein level through sponging miR-1299, thereby affecting the paclitaxel resistance in OC cells [13]. Therefore, circ_0001741 may be an important regulator for OC progression, and its role and mechanism in OC process deserve to be further revealed.

Our study aimed to explore the role and mechanism of circ_0001741 in OC progression. Through analysis, our study demonstrated the tumor-promoting properties of circ_0001741 in OC and pointed out the existed circ_0001741/miR-491-5p/PRSS8 axis in OC progression. This research may offer new ideas for the treatment of OC.

## Materials and methods

### Specimen

A total of 70 paired tumor tissues and adjacent normal tissues were obtained from OC patients at Yi chang Maternal and Child Health Hospital. Samples were confirmed by pathological diagnosis and were stored at −80 °C. Patients all signed informed consent forms, and the study was ratified by the Ethics Committee of Yi chang Maternal and Child Health Hospital in accordance with the Declaration of Helsinki. Inclusion criteria: age > 18 years; no participant had received anti-tumor therapies (such as radiotherapy or chemotherapy); signed written informed consent before the surgery; OC confirmed by pathological examination after surgery; completed clinical and pathological information. Exclusion criteria: patients with other cancers or a history of treatment for other cancers; patients with other ovarian diseases; severe complicated diseases or severe infectious diseases.

### Cell culture and transfection

OC cell lines (SKOV3, A2780, CAOV3, and OVCAR3) and normal ovarian epithelial cells (IOSE-80) (Biobw, Beijing, China) were cultured in DMEM containing 10% FBS and 1% penicillin/streptomycin solution (15,140,122; Gibco, Grand Island, NY, USA).

Small interfering RNA against circ_0001741 (si-circ_0001741), miR-491-3p mimic or inhibitor (anti-miR-491-3p), and negative controls (si-NC, miR-NC and anti-miR-NC) were synthesized by RiBoBio (Guangzhou, China). The pcDNA3.1 PRSS8 overexpression vector was structured by inserting the PCR products of PRSS8 into pcDNA3.1 vector. Cell transfection was performed using Lipo6000™ (C0526-1.5 mL; Beyotime, Shanghai, China). For lentiviral transduction in animal study, SKOV3 cells were infected by lentivirus circ_0001741 shRNA (sh-circ_0001741) or its control (sh-NC) in media containing 8 µg/ mL of polybrene. After 24 h, the stably infected SKOV3 cells were selected by 1 µg/mL of puromycin over 72 h.

### Quantitative real-time PCR (qRT-PCR)

As previously described [14], total RNAs were extracted using TRIzol reagent (15596026CN; Invitrogen, Carlsbad, CA, USA), and cDNA was obtained using PrimeScriptTM RT reagent kit (RR716; TaKaRa, Dalian, China). SYBR Green (RR820A; TaKaRa) was used for PCR amplification with specific primers (Table 1) and cDNA. Relative expression was calculated using 2^−ΔΔCt^ method with β-actin (for circ_0001741, PRSS8, and TNPO3) or U6 (for miR-491-5p) as internal control. Cytoplasmic & nuclear RNA purification kit (NGB-21000; Norgen, Ontario, Canada) was used to isolate nuclear and cytoplasm RNAs and then performed qRT-PCR. Besides, the extracted RNA was incubated with RNase R and then used for qRT-PCR to measure circ_0001741 and TNPO3 mRNA levels.

**Table 1:** Primer sequences used for qRT-PCR

Name	Primers (5ʹ–3ʹ)	
circ_0001741	Forward	CGGCGCACAGAAATTATAGA
	Reverse	CATGGTCTGTGCAGCAAAAT
miR-491-5p	Forward	GCCGAGAGTGGGGAACCCTTCC
	Reverse	CTCAACTGGTGTCGTGGA
PRSS8	Forward	CTATGAAGGCGTCCATGTGTG
	Reverse	AGTTACACGTCTCACGACTGAT
TNPO3	Forward	GAAGGAGCAAAGCCGACATTG
	Reverse	CCGGATCTGTAACAACTGGTC
β-actin	Forward	CTCCATCCTGGCCTCGCTGT
	Reverse	GCTGTCACCTTCACCGTTCC
U6	Forward	ATTGGAACGATACAGAGAAGATT
	Reverse	GGAACGCTTCACGAATTTG

### Cell counting kit 8 (CCK8) assay

As previously described [15], the digested OC cells were re-suspended and seeded into 96-well plates (2 × 10^3^ cells). CCK8 solution (C0037; Beyotime) was incubated with cells at each time point (0, 24, 48 and 72 h) for 2 h. The absorbance was examined at 450 nm using a microplate reader.

### Edu assay

Edu In Vitro Kit (C10310-1; RiBoBio) was used for measuring cell proliferation according to the previous study described [16]. OC cells were inoculated into 96-well plates (2 × 10^4^ cells), and then incubated with Edu solution and DAPI solution. Edu positive (Edu^+^) cells were counted under fluorescent microscope with ImageJ software.

### Flow cytometry

As previously described [17], OC cells (2 × 10^5^ cells) were collected and then stained with Annexin V-FITC and PI solution (A211; Vazyme, Nanjing, China). Cell apoptosis rate was analyzed by FACScalibur flow cytometer with CellQuest Pro software.

### Cell migration and invasion assays

Cell suspended with serum-free medium was added to the upper of Transwell chambers (5 × 10^5^ cells/well) pre-coated with or without matrigel (356,255; Corning Inc., Corning, NY, USA), while completed medium was added into lower chamber. After incubation for 24 h, cells were fixed with 4% paraformaldehyde and stained by crystal violet (C0121-100 mL; Beyotime), and the migrated cells and invaded cells were counted with a microscope with ImageJ software.

### Western blot

Western blot analysis was performed as previously described [15]. Briefly, proteins were extracted by RIPA buffer (P0013B; Beyotime), separated by SDS-PAGE gel and transferred to PVDF membranes. After blockage, membranes were incubated with specific primary antibodies (Abcam, Cambridge, MA, USA) including anti-Bax (1:1000, ab53154), anti-Bcl-2 (1:1000, ab196495), anti-Snail (1:1000, ab216347), anti-MMP9 (1:1000, ab38898), anti-N-cadherin (1:1000, ab245117), anti-PRSS8 (1:1000, ab200736), anti-β-actin (1:2000, ab8227) at 4 °C overnight, and then incubated with secondary antibody (1:2000) at room temperature for 1 h. Then, membrane was treated with ECL luminescent solution (P0018S; Beyotime), and image grayscale value was analyzed by Image J software.

### Dual-luciferase reporter assay

As previously described [14], circ_0001741 containing miR-491-5p binding sequence (circ_0001741-WT) and its mutant sequence (circ_0001741-MUT), PRSS8 3'UTR sequence (PRSS8 3'UTR-WT) and its mutant sequence (PRSS8 3'UTR-MUT) were inserted into pmirGLO vector. The vector was co-transfected with miR-NC/miR-491-5p into SKOV3 and A2780 cells. After 48 h, cells were assayed for detecting luciferase activity.

### RIP assay

RIP test was performed according to the Magna RIP kit (17–700; Millipore, Billerica, MA, USA) instructions. As previously described [18], OC cells were lysed with RIP buffer, and then the cell lysates were incubated with magnetic beads and anti-IgG or anti-Ago2 overnight. Immunoprecipitated RNA was collected to detect circ_0001741, miR-491-5p and PRSS8 enrichments by qRT-PCR.

### Animal experiments

According to the previous study [18], BALB/c nude mice were subcutaneously injected with 1 × 10^7^ SKOV3 cells stably infected with lentiviral sh-circ_0001741 or sh-NC (N = 6). Tumor volumes were measured weekly after cell injection. Mice were sacrificed by cervical dislocation under 2% isoflurane anesthesia at 35 days, and tumors were excised, photographed, and weighed. Animal research was approved by the Animal Ethics Committee of Yi chang Maternal and Child Health Hospital and was performed in compliance with the ARRIVE guidelines.

### Statistical analysis

GraphPad Prism 7.0 analysis software was used to analyze the data, and the data were expressed as mean ± SD. Values between groups were analyzed using Student's *t*-test and ANOVA. *P* < 0.05 was considered statistically significant.

## Results

### Circ_0001741 was upregulated in OC

**Fig. 1:**
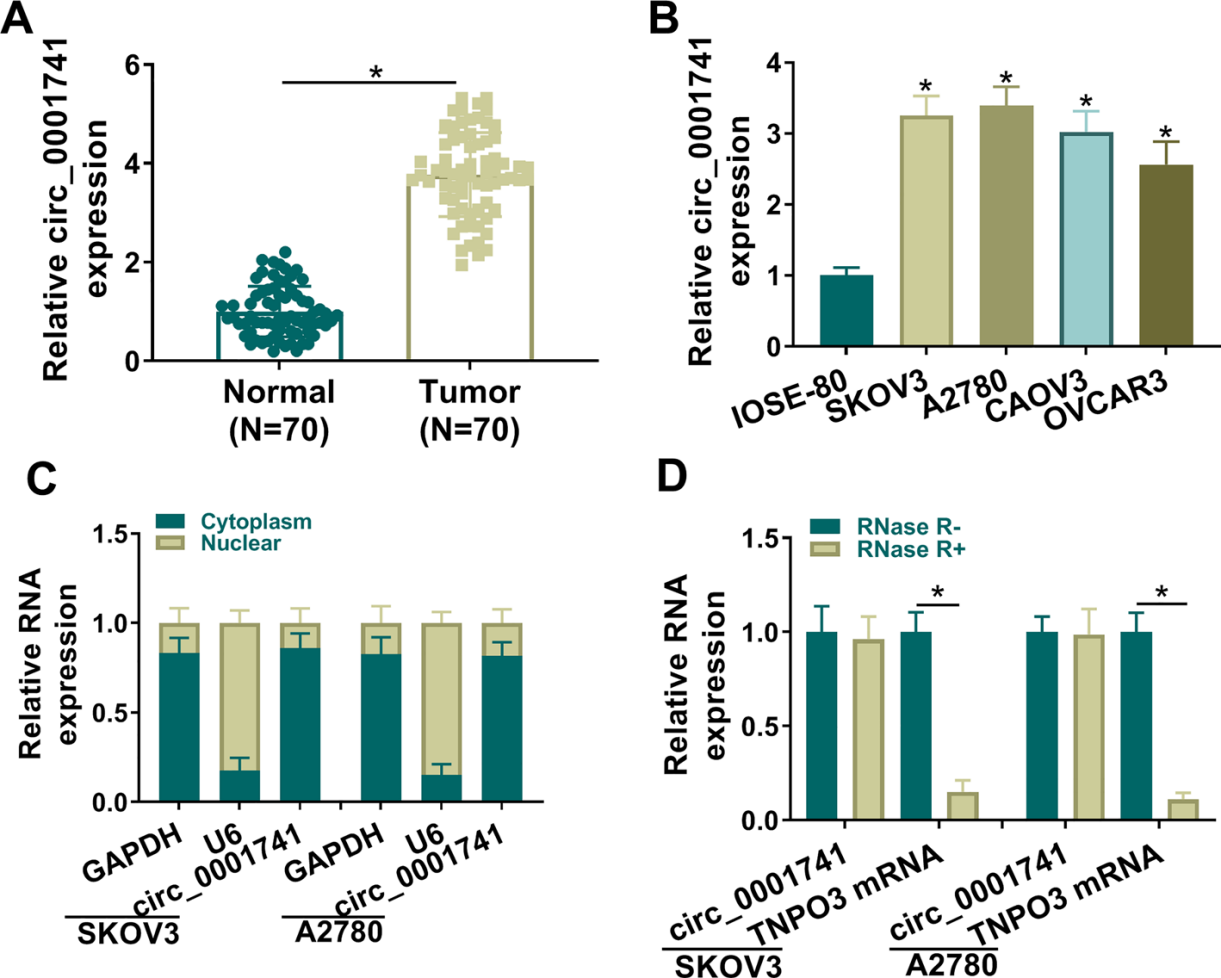
Circ_0001741 was upregulated in OC. **A** qRT-PCR measured circ_0001741 expression in tumor and normal samples. n = 70. **B** qRT-PCR measured circ_0001741 levels in OC cells (SKOV3, A2780, CAOV3, and OVCAR3) and normal ovarian epithelial cells (IOSE-80). **C** qRT-PCR measured circ_0001741 levels in SKOV3 and A2780 cells. **D** After RNase R digestion, qRT-PCR was performed to detect circ_0001741 and TNPO3 mRNA levels in SKOV3 and A2780 cells. A, Paired *t*-test; B, Ordinary one-way ANOVA; C-D, 2way ANOVA. *P < 0.05

Through qRT-PCR, we found that circ_0001741 expression was higher in OC tissues and cells (Fig. 1A, B). Then, circ_0001741 was found to be mainly present in the cytoplasm of SKOV3 and A2780 cells (Fig. 1C). Besides, circ_0001741 could resist to RNase R digestion compared to linear RNA TNPO3 (Fig. 1D), confirming the circular feature of circ_0001741.

### Circ_0001741 knockdown inhibited growth and metastasis in OC cells

**Fig. 2:**
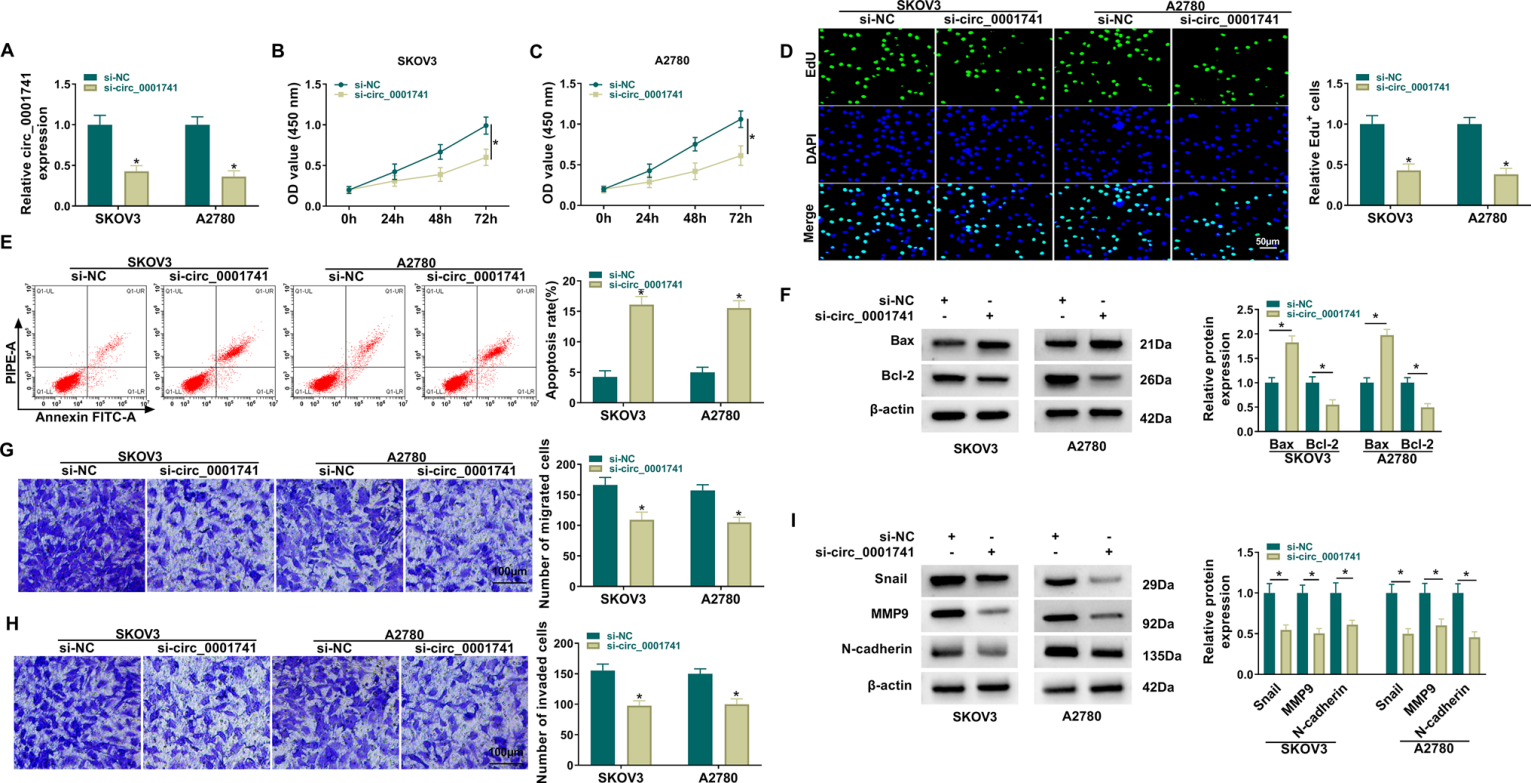
Circ_0001741 knockdown inhibited OC cell progression. SKOV3 and A2780 cells were transfected with si-NC or si-circ_0001741. **A** qRT-PCR was used to detect transfection efficiency in SKOV3 and A2780. CCK8 (**B**-**C**) and Edu staining (magnification 200 ×) (**D**) were used to detect SKOV3 and A2780 proliferation. **E** Flow cytometry was used to detect SKOV3 and A2780 apoptosis. **F** Western blot was performed to examine Bax and Bcl-2 expression. **G**, **H** Transwell was employed to test SKOV3 and A2780 migration and invasion (magnification 100 ×). **I** Western blot was used to measure Snail, MMP9 and N-cadherin expression. A and D-I, Unpaired *t*-test; B-C; 2way ANOVA. *P < 0.05

To further investigate the role of circ_0001741 in OC cell progression, OC cells were transfected with si-circ_0001741. The addition of si-circ_0001741 effectively reduced the expression of circ_0001741 (Fig. 2A). Silencing circ_0001741 repressed cell proliferation (Fig. 2B–D) and promoted apoptosis (Fig. 2E). Western blot results showed that circ_0001741 knockdown increased Bax expression and inhibited Bcl-2 expression (Fig. 2F). Circ_0001741 knockdown inhibited OC cell migration and invasion, as well as the protein levels of Snail, MMP9, and N-cadherin (Fig. 2G–I). These data suggested that circ_0001741 might promote OC cell progression.

### Circ_0001741 targeted miR-491-5p

**Fig. 3:**
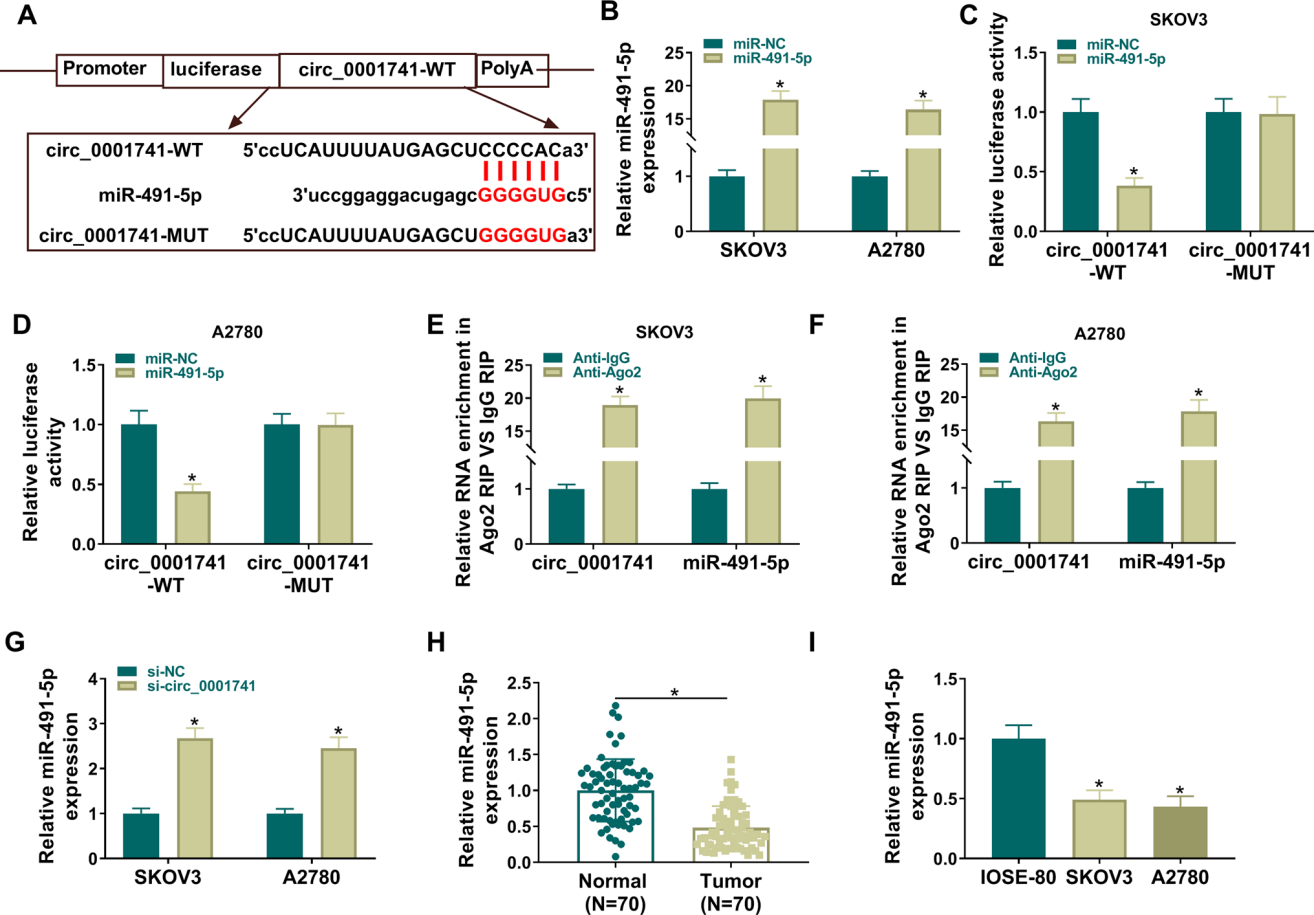
Circ_0001741 targeted miR-491-5p. **A** Predicted complementary sites of circ_0001741 with miR-491-5p, and mutants of seeded sites. **B** qRT-PCR detection of miR-491-5p expression in SKOV3 and A2780 transfected with miR-491-5p mimic. **C**, **D** Wild-type (WT) or mutant (MUT) reporters were co-transfected with miR-NC or miR-491-5p into SKOV3 and A2780 cells, and luciferase activity was measured 48 h post-transfection. **E**, **F** RIP assay was performed to detect the interaction between circ_0001741 and miR-491-5p. **G** qRT-PCR analysis was used to detect miR-491-5p expression in cells transfected with si-NC or si-circ_0001741. **H** qRT-PCR was performed to determine miR-491-5p expression in tumor and normal samples. n = 70. **I** qRT-PCR was used to measure miR-491-5p level in OC cells (SKOV3 and A2780) and normal ovarian epithelial cells (IOSE-80). B-D and G, Unpaired *t*-test; E–F; Welch's *t*-test; H, Paired *t*-test; I, Ordinary one-way ANOVA. *P < 0.05

To further determine the mechanism of circ_0001741 in regulating OC, we identified its target miRNAs. Through the prediction of circbank database (http://www.circbank.cn/searchMiRNA.html), we found that circ_0001741 had a target binding sites with miR-491-5p (Fig. 3A). The overexpression efficiency of miR-491-5p mimic was confirmed by qRT-PCR (Fig. 3B). MiR-491-5p overexpression in SKOV3 and A2780 cells significantly inhibited luciferase activity in the circ_0001741-WT group (Fig. 3C, D), and circ_0001741 and miR-491-5p were highly enriched by Anti-Ago2 (Fig. 3E, F). These data confirmed the interaction between circ_0001741 and miR-491-5p. In addition, miR-491-5p expression was elevated in SKOV3 and A2780 transfected with si-circ_0001741 (Fig. 3G). Moreover, we confirmed that miR-491-5p expression was downregulated in OC tissues and cells (Fig. 3H, I).

### MiR-491-5p inhibitor reversed the effect of circ_0001741 knockdown on OC cell progression

**Fig. 4:**
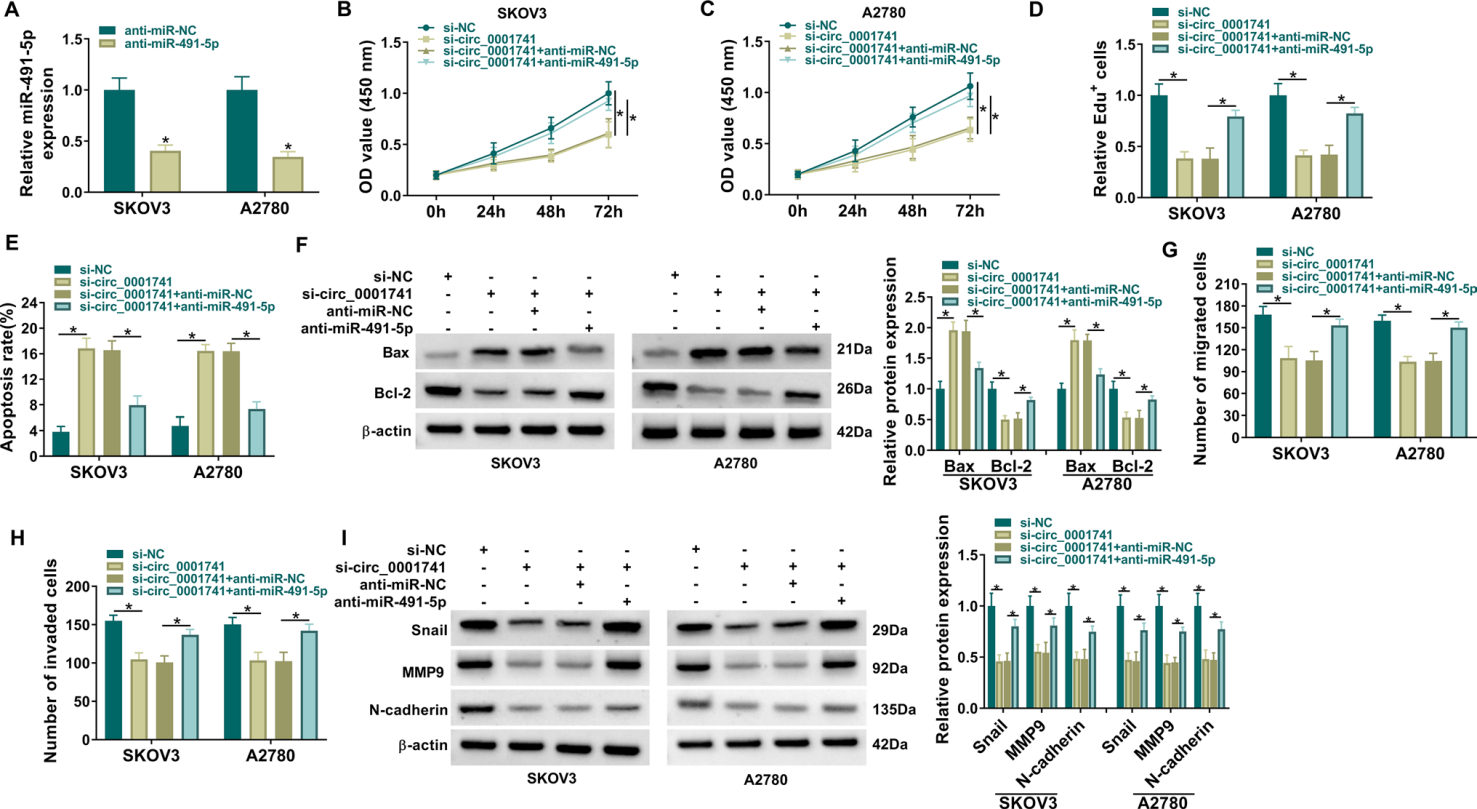
MiR-491-5p downregulation reversed the effect of circ_0001741 knockdown on OC cell progression. **A** qRT-PCR assay was used for assessing the transfection efficiency of anti-miR-491-5p in SKOV3 and A2780. (B-I) SKOV3 and A2780 cells were transfected with si-NC, si-circ_0001741, si-circ_0001741 + anti-miR-NC or si-circ_0001741 + anti-miR-491-5p. **B**–**D** CCK8 and Edu staining assays were performed for measuring SKOV3 and A2780 proliferation. **E** Flow cytometry was performed to test SKOV3 and A2780 apoptosis. **F** Western blot was used to detect Bax and Bcl-2 expression. **G**, **H** Transwell was utilized to examine SKOV3 and A2780 migration and invasion. **I** Western blot was employed to measure Snail, MMP9 and N-cadherin expression. A, Unpaired *t*-test; B-C; 2way ANOVA; D-I, Ordinary one-way ANOVA. *P < 0.05

Further analysis was performed to further investigate the interaction of circ_0001741 and miR-491-5p on the biological behavior of OC cells. The transfection of anti-miR-491-5p markedly reduced miR-491-5p expression in SKOV3 and A2780 (Fig. 4A). MiR-491-5p inhibitor attenuated circ_0001741 knockdown-mediated proliferation inhibition (Fig. 4B–D and Supplementary Fig. 1A). In addition, the promotion effect of circ_0001741 silencing on cell apoptosis was reversed by miR-491-5p inhibitor (Fig. 4E and Supplementary Fig. 1B). Also, miR-491-5p inhibitor reversed the effect of circ_0001741 silencing on Bax and Bcl-2 levels (Fig. 4F). MiR-491-5p inhibitor abolished the effect of circ_0001741 silencing-mediated the inhibition on cell migration and invasion (Fig. 4G, H and Supplementary Fig. 1C, D). Besides, miR-491-5p reversed the si-circ_0001741-mediated the reduction on Snail, MMP, and N-cadherin expression (Fig. 4I). All data confirmed that circ_0001741 regulated OC cell progression by sponging miR-491-5p.

### MiR-491-5p targeted PRSS8

**Fig. 5:**
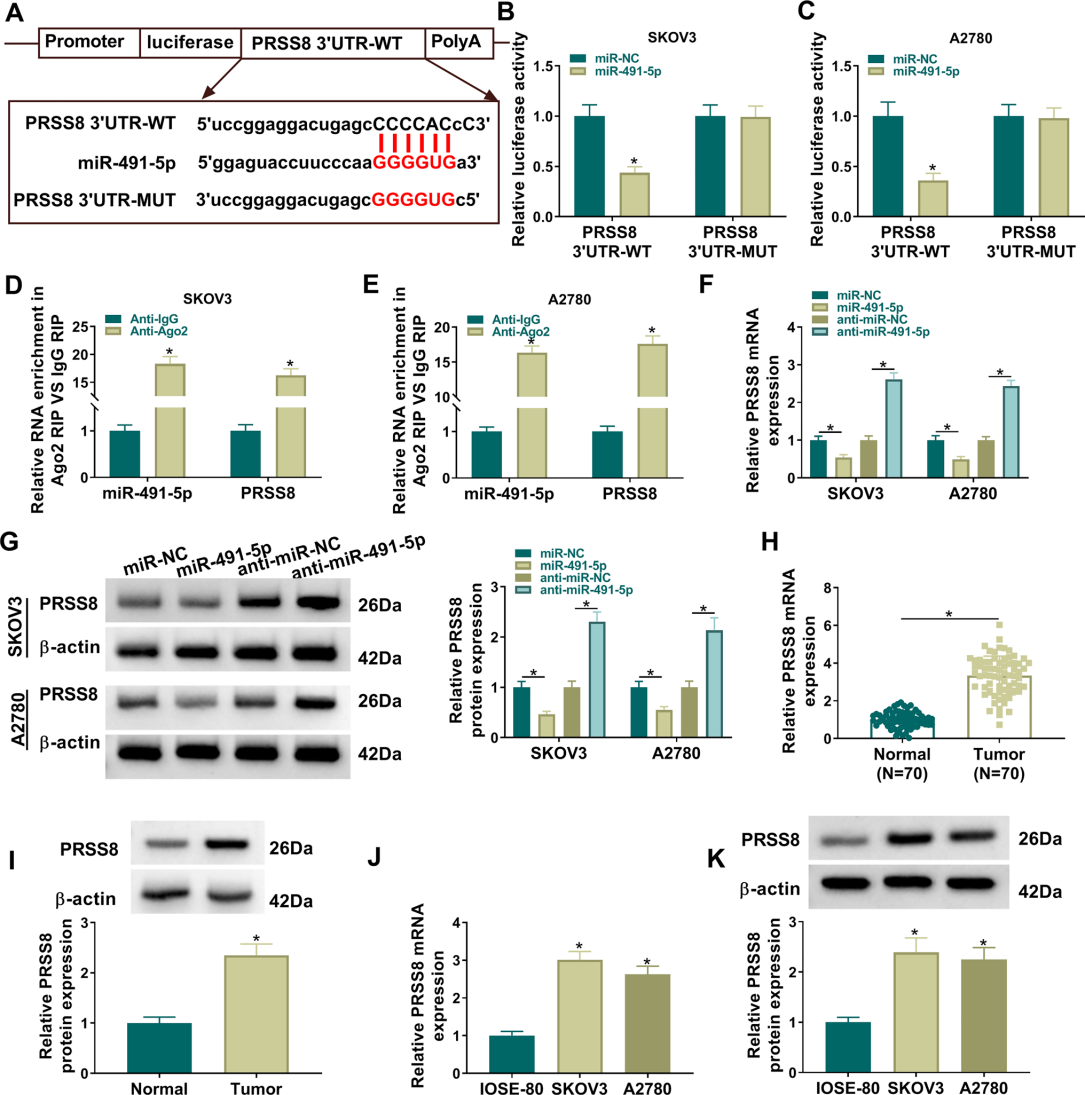
miR-491-5p targeted PRSS8. **A** Predicted complementary sites for miR-491-5p and PRSS8, and mutants at the seeded sites. **B**, **C** Wild-type (WT) or mutant (MUT) reporter constructs were co-transfected with miR-NC or miR-491-5p into SKOV3 and A2780 cells, and luciferase activity was measured 48 h post-transfection. **D**, **E** The binding relationship between miR-491-5p and PRSS8 in SKOV3 and A2780 was detected by RIP assay. **F**, **G** qRT-PCR and western blot were performed to detect the regulatory of miR-491-5p on PRSS8 expression. **H**, **I** qRT-PCR and western blot were performed to determine PRSS8 mRNA and protein expression in tumor and normal samples. n = 70. **J**, **K** qRT-PCR and western blot were used to measure PRSS8 mRNA and protein levels in OC cells (SKOV3 and A2780) and normal ovarian epithelial cells (IOSE-80). **B**, **C** and **F**, **G**, Unpaired *t*-test; **D**, **E**; Welch's *t*-test; **H**, **I**, Paired *t*-test; **J**, **K**, Ordinary one-way ANOVA. *P < 0.05

Due to miRNAs usually function by binding to the mRNA 3'UTR [19], we next identified the downstream targets of miR-491-5p. Also, ENCORI database (https://rnasysu.com/encori/index.php) predicted that miR-491-5p had a target binding site to PRSS8 3’UTR (Fig. 5A). MiR-491-5p overexpression reduced luciferase activity in the PRSS8 3'UTR-WT group (Fig. 5B, C), and miR-491-5p and PRSS8 were highly enriched by Anti-Ago2 (Fig. 5D, E), confirming the interaction between miR-491-5p and PRSS8. PRSS8 mRNA and protein levels could be promoted by miR-491-5p deletion and suppressed by miR-491-5p mimic (Fig. 5F, G). PRSS8 mRNA and protein levels were enhanced in OC tissues and cells (Fig. 5H–K). These data confirmed that PRSS8 was targeted by miR-491-5p.

### PRSS8 overexpression restored the effects of miR-491-5p mimics on OC cell progression

**Fig. 6:**
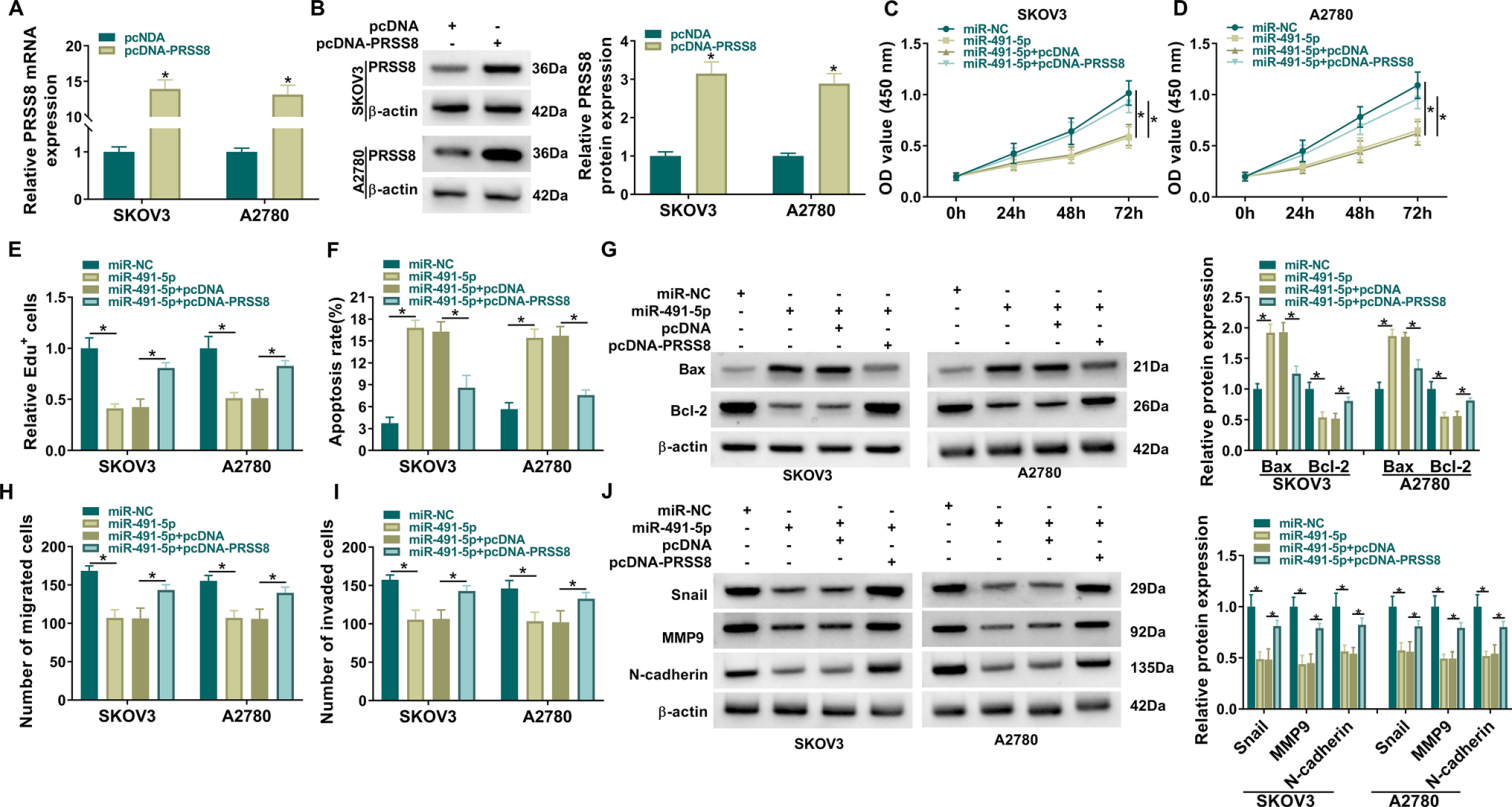
PRSS8 overexpression reverses the effect of miR-491-5p mimics on OC cell progression. **A**, **B** qRT-PCR and western blot were employed to detect PRSS8 mRNA and protein expression in OC cells transfected with pcDNA or pcDNA-PRSS8. **C**–**J** SKOV3 and A2780 cells were transfected with miR-NC, miR-491-5p, miR-491-5p + pcDNA or miR-491-5p + pcDNA-PRSS8. **C**–**E** CCK8 and Edu staining were employed to detect SKOV3 and A2780 proliferation. **F** Flow cytometry was used to examine SKOV3 and A2780 apoptosis. **G** Western blot was performed to test Bax and Bcl-2 expression. **H**–**I** Transwell was employed to detect SKOV3 and A2780 migration and invasion. **J** Western blot was used to measure Snail. MMP9 and N-cadherin expression. A-B, Welch's *t*-test; C-D; 2way ANOVA; E-J, Ordinary one-way ANOVA. *P < 0.05

To further explore whether miR-491-5p regulated OC cell progression by targeting PRSS8, we performed rescue experiments. The detection of PRSS8 mRNA and protein expression confirmed the transfection efficiency of pcDNA-PRSS8 (Fig. 6A, B). Overexpression of miR-491-5p inhibited OC cell proliferation and promoted apoptosis, which were reversed by PRSS8 upregulation (Fig. 6C–G and Supplementary Fig. 2A, B). Increased PRSS8 expression attenuated the inhibition effect of miR-491-5p on OC cell migration and invasion (Fig. 6H, I and Supplementary Fig. 2C, D). PRSS8 overexpression also abrogated the inhibitory effects of miR-491-5p on Snail, MMP9, and N-cadherin levels (Fig. 6J). It was confirmed that miR-491-5p suppressed OC cell development by regulating PRSS8.

### Circ_0001741 regulated PRSS8 expression by targeting miR-491-5p

**Fig. 7:**
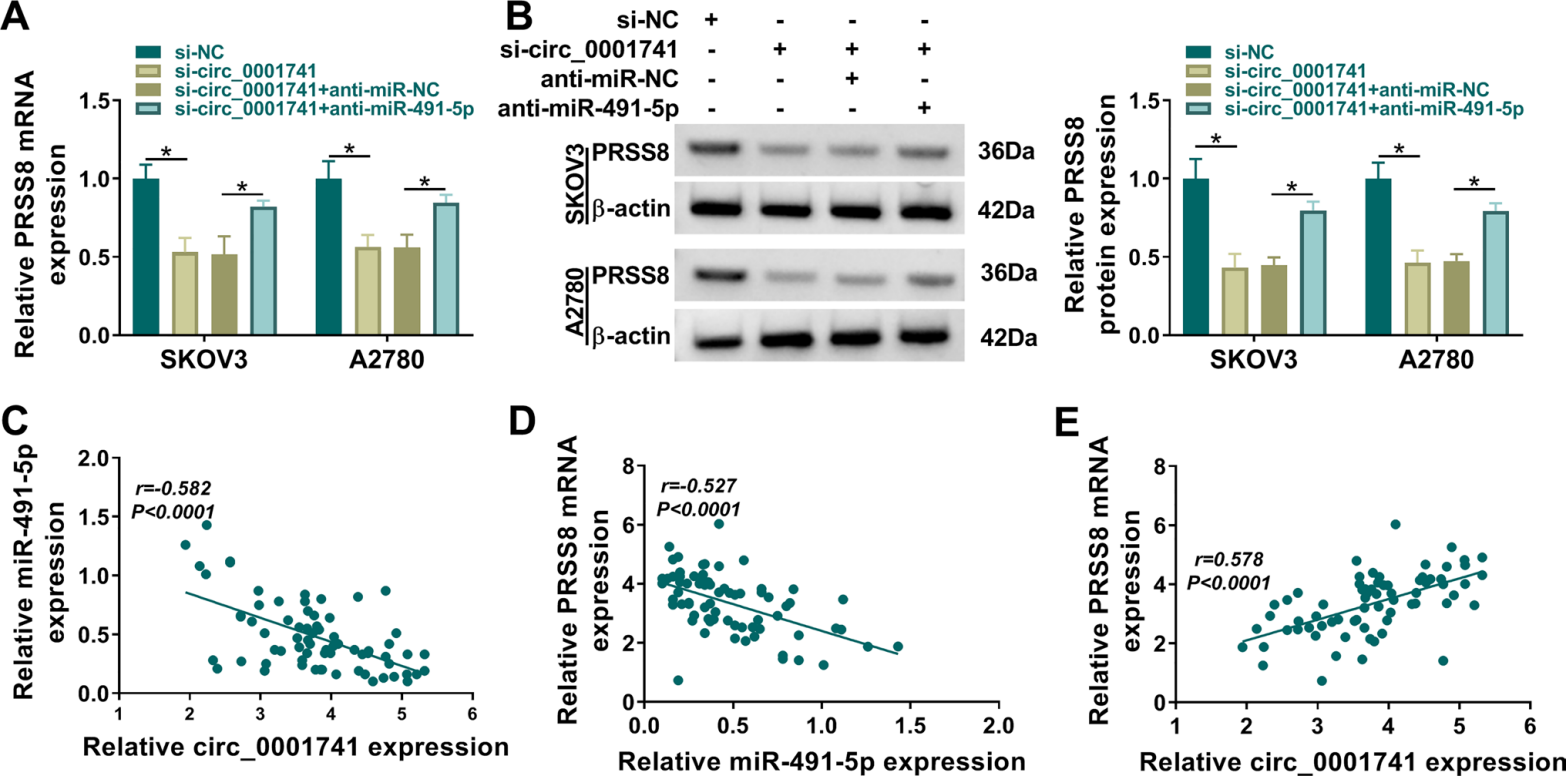
Circ_0001741 regulated PRSS8 expression by targeting miR-491-5p**.** **A**, **B** qRT-PCR and western blot were used to measure PRSS8 mRNA and protein levels in OC cells. **C**–**E** Linear relationship between circ_0001741, miR-491-5p and PRSS8 mRNA expression in OC tissues was analyzed by Pearson's correlation coefficient. A-B, Ordinary one-way ANOVA. *P < 0.05

Subsequently, we explored whether circ_0001741 regulated PRSS8 expression by sponging miR-491-5p in OC cells. Notably, the reduction of circ_0001741 expression led to a decrease in PRSS8 levels, and this effect was reversed by miR-491-5p downregulation (Fig. 7A, B). In OC tissues, we found that miR-491-5p expression was negatively correlated with circ_0001741 expression and PRSS8 mRNA expression (Fig. 7C, D), while circ_0001741 expression was positively correlated with PRSS8 expression (Fig. 7E). All data suggested that circ_0001741 regulated PRSS8 expression by sponging miR-491-5p.

### Circ_0001741 knockdown suppressed OC tumorigenesis

**Fig. 8:**
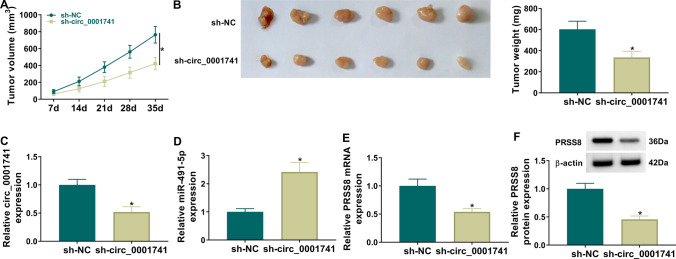
Impacts of hsa_circ_0001741 on tumorigenesis in xenograft model. **A** Growth curves of xenogeneic tumors were obtained by injecting sh-circ_0001741/sh-NC-transfected SKOV3 cells into nude mice (N = 6 per group). **B** Tumor weights were measured. **C**–**F** qRT-PCR and western blot were performed to detect circ_0001741, miR-491-5p and PRSS8 levels in mice tumor tissues. A, 2way ANOVA; B-F, Unpaired *t*-test. *P < 0.05

Next, a nude mouse model was established to explore the role of circ_0001741 knockdown in vivo. The tumor volume and weight were significantly reduced in the sh-circ_0001741 group (Fig. 8A, B). Circ_0001741 and PRSS8 levels were inhibited, while miR-491-5p expression was enhanced in the sh-circ_0001741 group (Fig. 8C–F). In conclusion, circ_0001741 silencing inhibited OC tumor growth by regulating miR-491-5p/PRSS8 pathway.

## Discussion

Many researches have demonstrated that circRNAs are aberrantly expressed in many malignant cancers and have a significant role in tumorigenesis [20–22]. Here, we explored the role and mechanism of circ_0001741 in OC progression. Studies had suggested that circ_0001741 had elevated expression in OC tissues and was associated with the paclitaxel resistance of OC [13]. Consistent with this study, our data also confirmed the high expression of circ_0001741 in OC tissues. Further analysis revealed that silencing of circ_0001741 inhibited OC cell growth, migration, and invasion in vitro, as well as suppressed OC tumor growth in vivo. These results fully confirmed that circ_0001741 played the oncogenic role in OC.

Previous research indicates that circRNAs play biological functions by sponging miRNAs [23]. Here, we confirmed that miR-491-5p could be sponged by circ_0001741. It had been reported that miR-491-5p could suppress esophageal squamous cell carcinoma cell stemness and metastasis [24]. Besides, miR-491-5p had been found to suppress glioblastoma multiform cell growth and autophagy [25]. In OC-related study, miR-491-5p might play tumor suppressor to participate in OC progression [26, 27]. Consistent with previous study, our data showed that miR-491-5p overexpression suppressed OC cell growth and metastasis, confirming the anti-tumor role of miR-491-5p in OC. Further analysis showed that circ_0001741 negatively regulated miR-491-5p expression in vitro and in vivo, and anti-miR-491-5p could reverse the inhibition effect of si-circ_0001741 on OC cell growth and metastasis. These data fully confirmed that circ_0001741 sponged miR-491-5p to promote OC progression.

PRSS8 is thought to play different roles in different cancers. Studies had shown that PRSS8 served as a tumor suppressor to regulate colon cancer process [28]. On the contrary, PRSS8 had been shown to be overexpressed in OC tissues, and could promote OC cell proliferation and migration [29]. Besides, PRSS8 was expressed at more than 100-fold higher levels in OC than in normal or benign ovarian lesions [30], which was also confirmed in our study. Next, miR-491-5p was found to target the PRSS8 3'UTR and thereby down-regulating PRSS8 mRNA and protein expression. Functional experiments suggested that PRSS8 overexpression attenuated the suppression effect of miR-491-5p overexpression on the malignant phenotype of OC cells, confirming that miR-491-5p targeted PRSS8 to inhibit OC progression. Additionally, we found that there had a positively regulation of circ_0001741 on PRSS8, which further supported the existence of the circ_0001741/miR-491-5p/PRSS8 axis in OC. Our study focused on PRSS8 as a downstream target of miR-491-5p. Of course, we do not exclude that there are other possible targets of miR-491-5p mediating OC progression, which need to be further explored in the future.

In summary, this study confirmed that circ_0001741 sponged miR-491-5p to positively regulate PRSS8, thereby accelerating the malignant phenotype of OC cells. This experiment revealed the interaction between circ_0001741, miR-491-5p, and PRSS8 in OC progression, and provided a potential therapeutic target for OC.

## Supplementary Information

Additional file1

Additional file2

Additional file3

## Data Availability

The datasets generated during the current study are available from the corresponding author on reasonable request.
