# Supplementary material for: Circ_0001741 exerts as a tumor promoter in ovarian cancer through the regulation of miR-491-5p/PRSS8 axis
Source: Discov Oncol. 2024 Nov 11;15:643. doi: 10.1007/s12672-024-01474-3 (PMC11554978; doi:10.1007/s12672-024-01474-3)
Supplement: Supplementary file 1 — Additional file1 [file 12672_2024_1474_MOESM1_ESM.pdf]

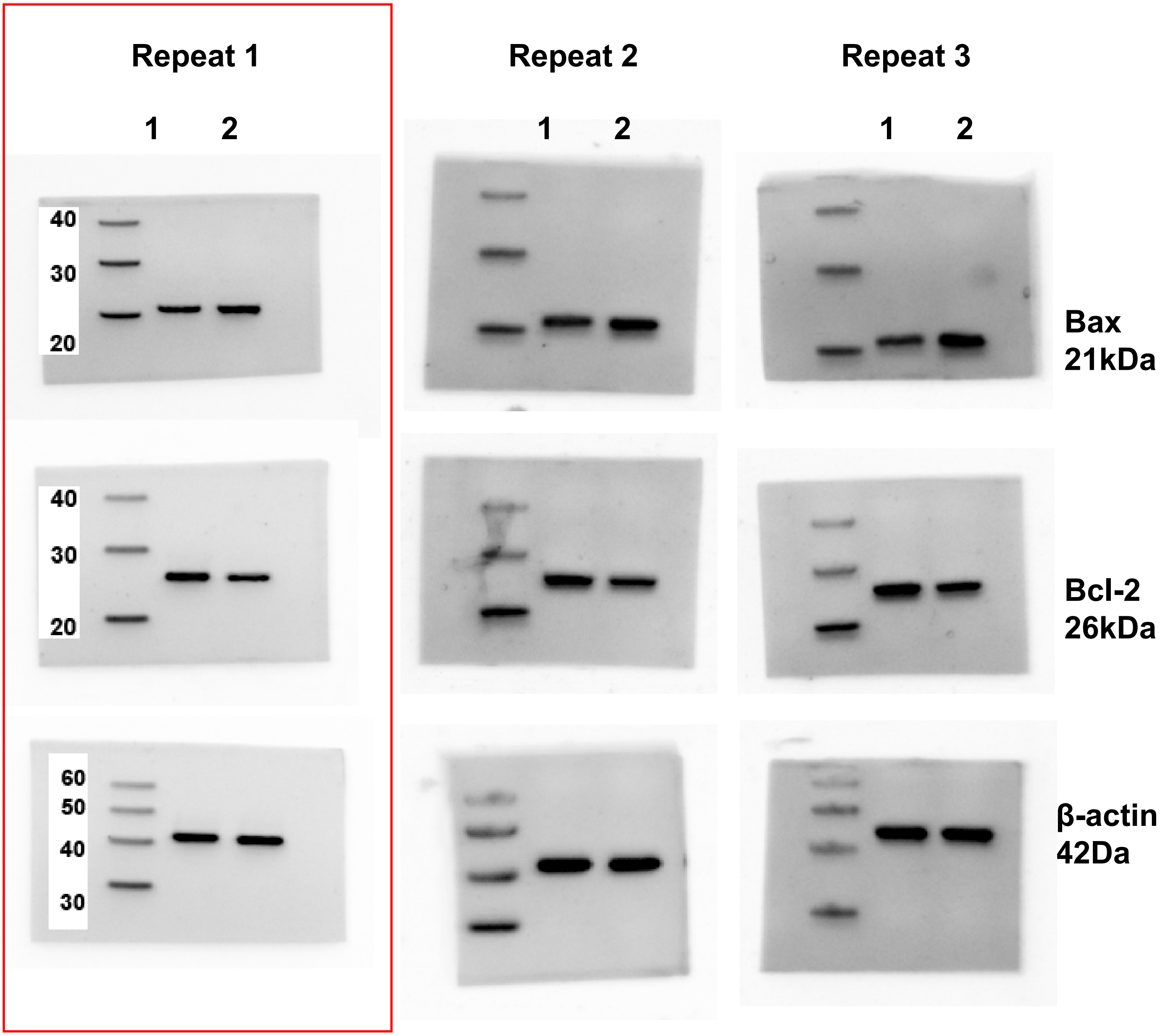

Fig 2F
SKOV3

1 si-NC
2 si-circ_0001741

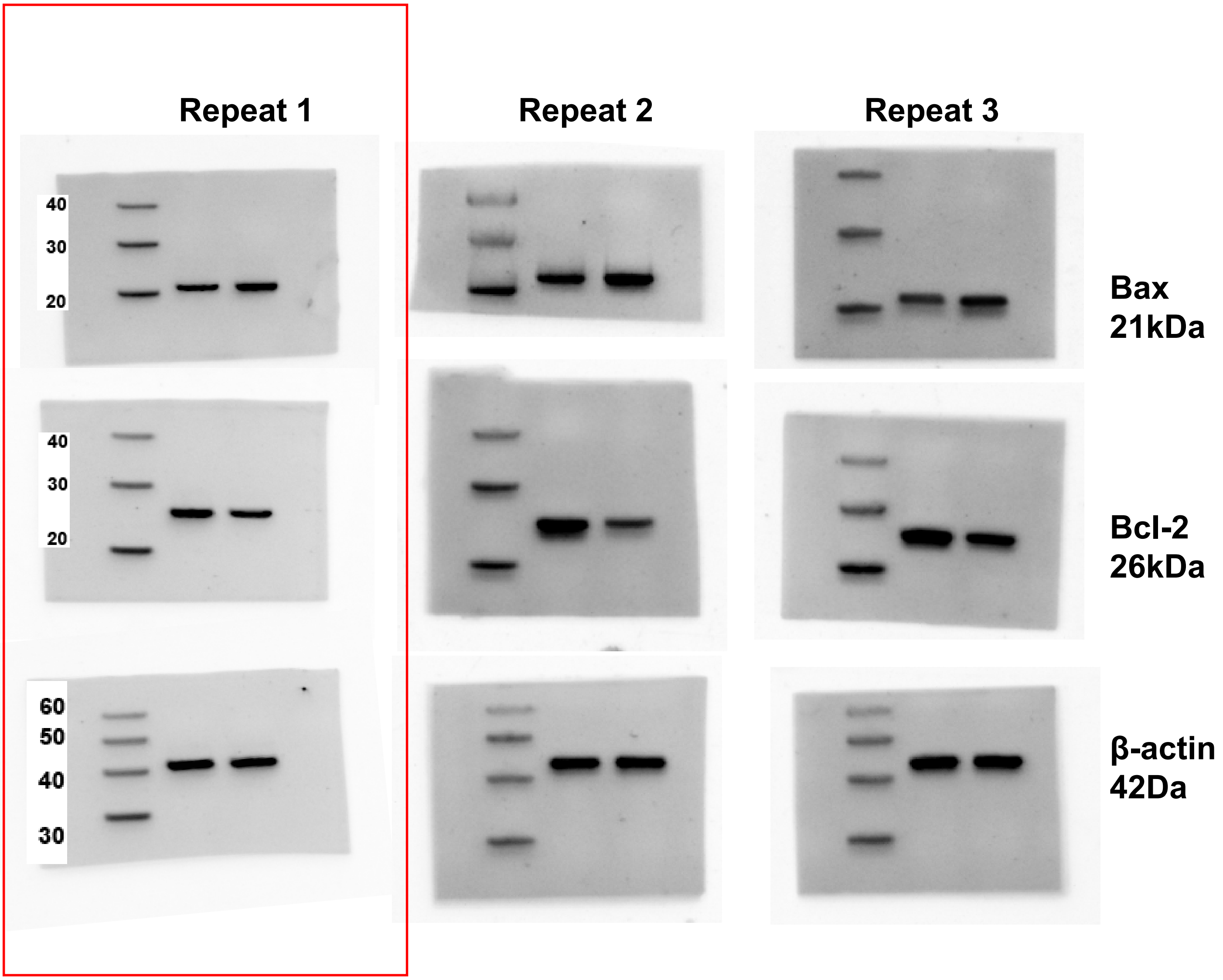

Fig 2F 1 si-NC
A2780 2 si-circ_0001741

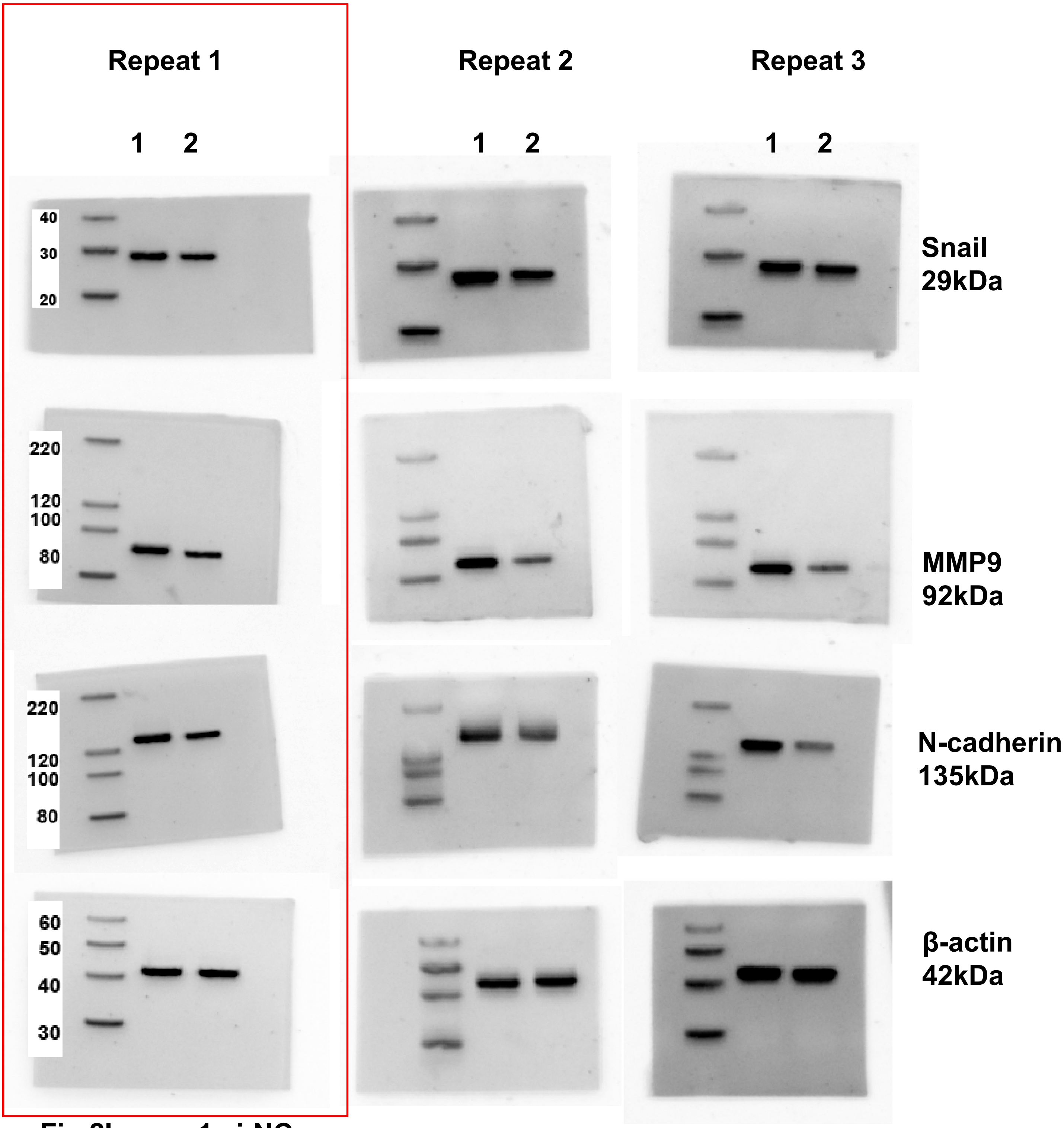

Fig 2I
SKOV3

1 si-NC
2 si-circ_0001741

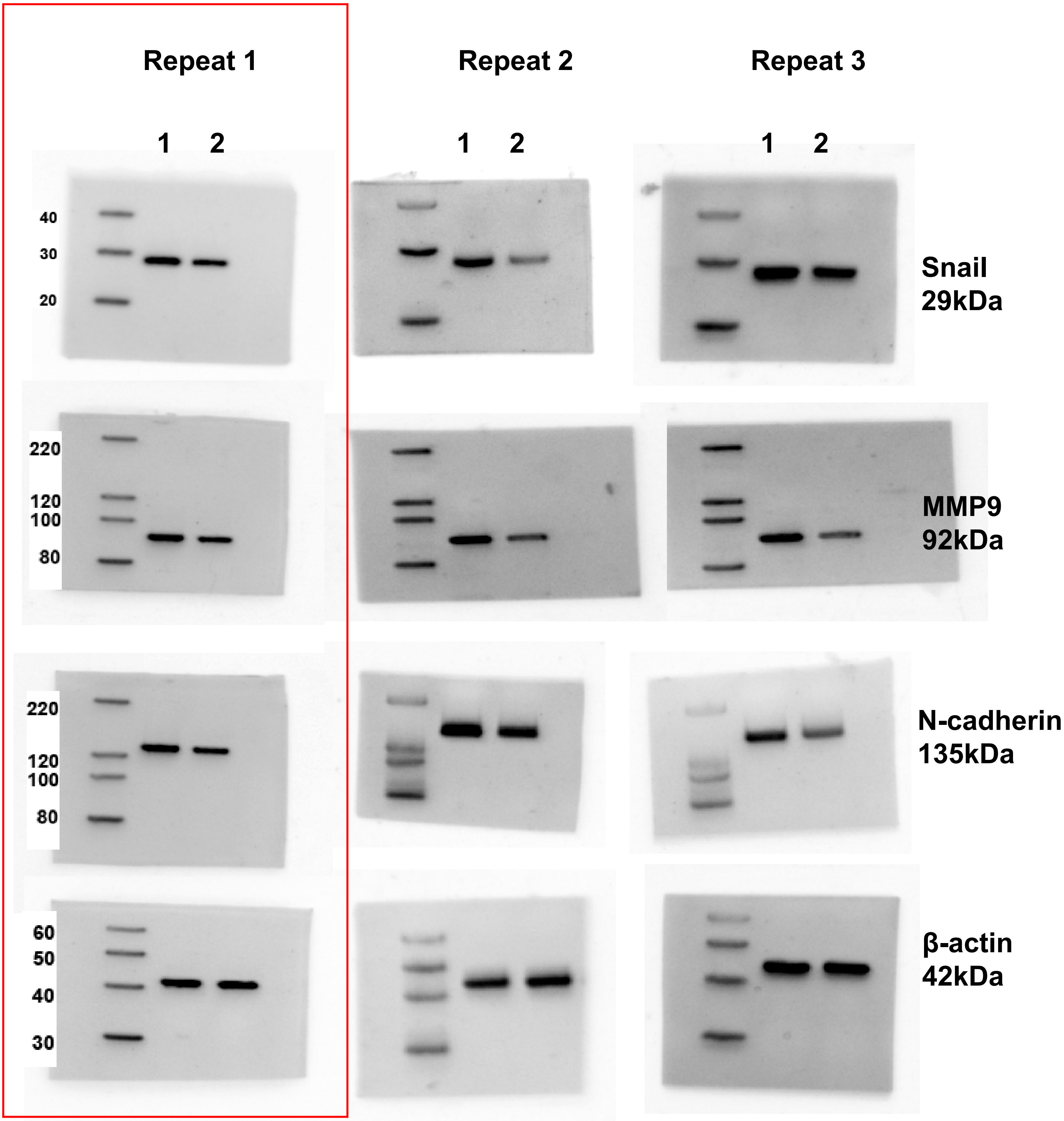

Fig 2I
A2780

1 si-NC
2 si-circ_0001741

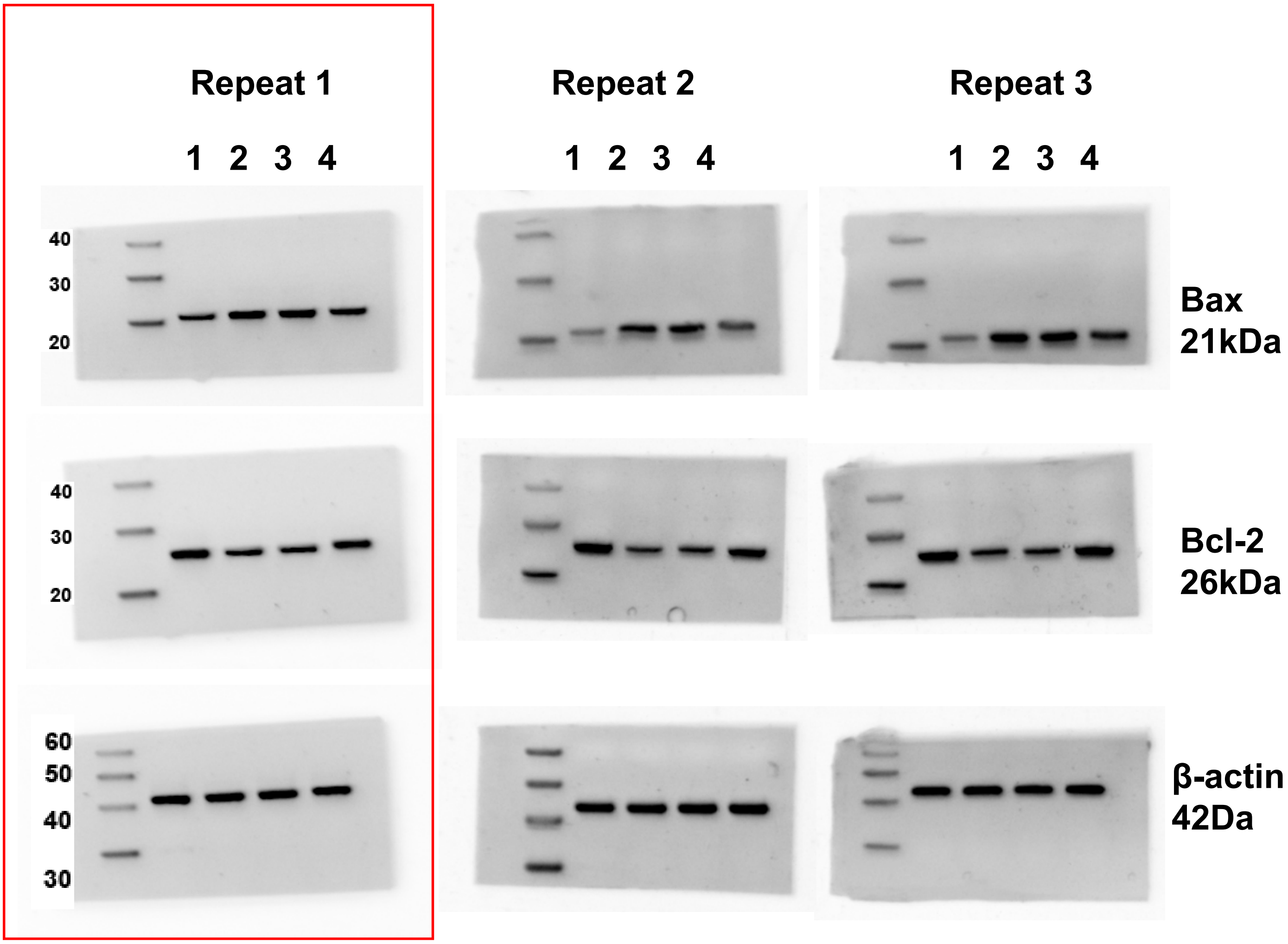

Fig 4F 1 si-NC
SKOV3 2 si-circ_0001741
3 si-circ_0001741+anti-miR-NC
4 si-circ_0001741+anti-miR-491-5p

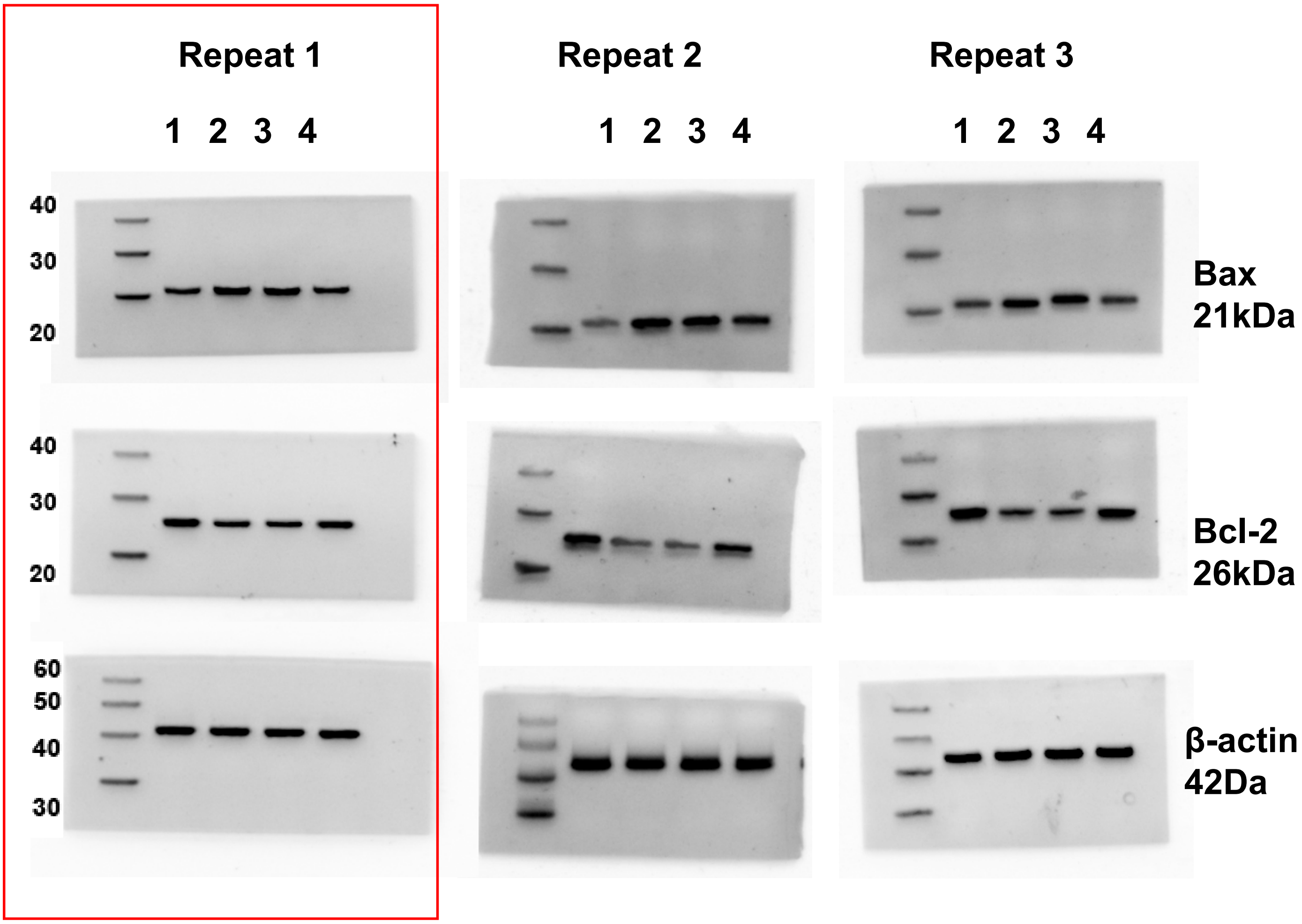

Fig 2F 1 si-NC
A2780 2 si-circ_0001741
3 si-circ_0001741+anti-miR-NC
4 si-circ_0001741+anti-miR-491-5p

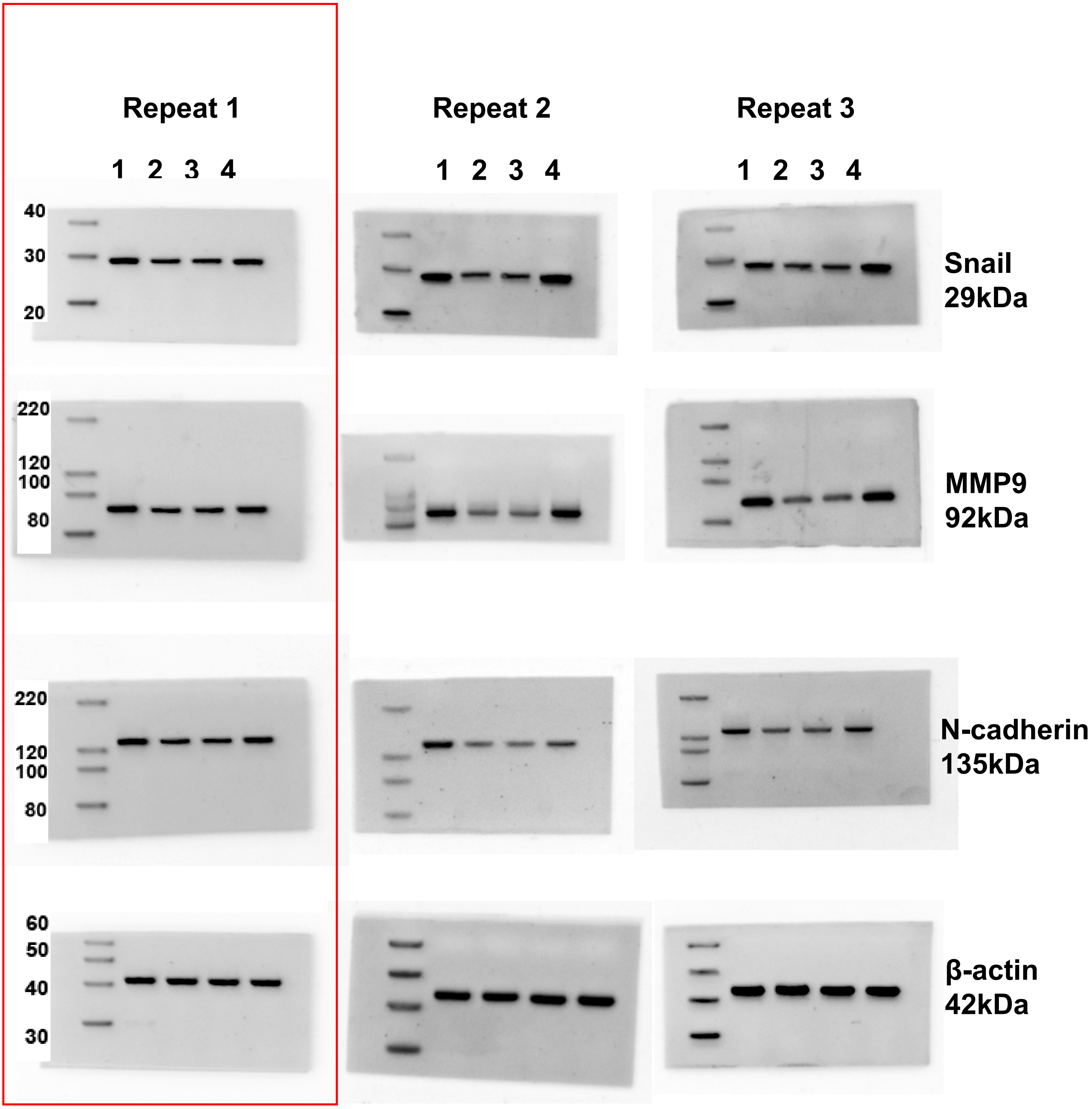

Fig 2I
SKOV3

1 si-NC
2 si-circ_0001741
3 si-circ_0001741+anti-miR-NC
4 si-circ_0001741+anti-miR-491-5p

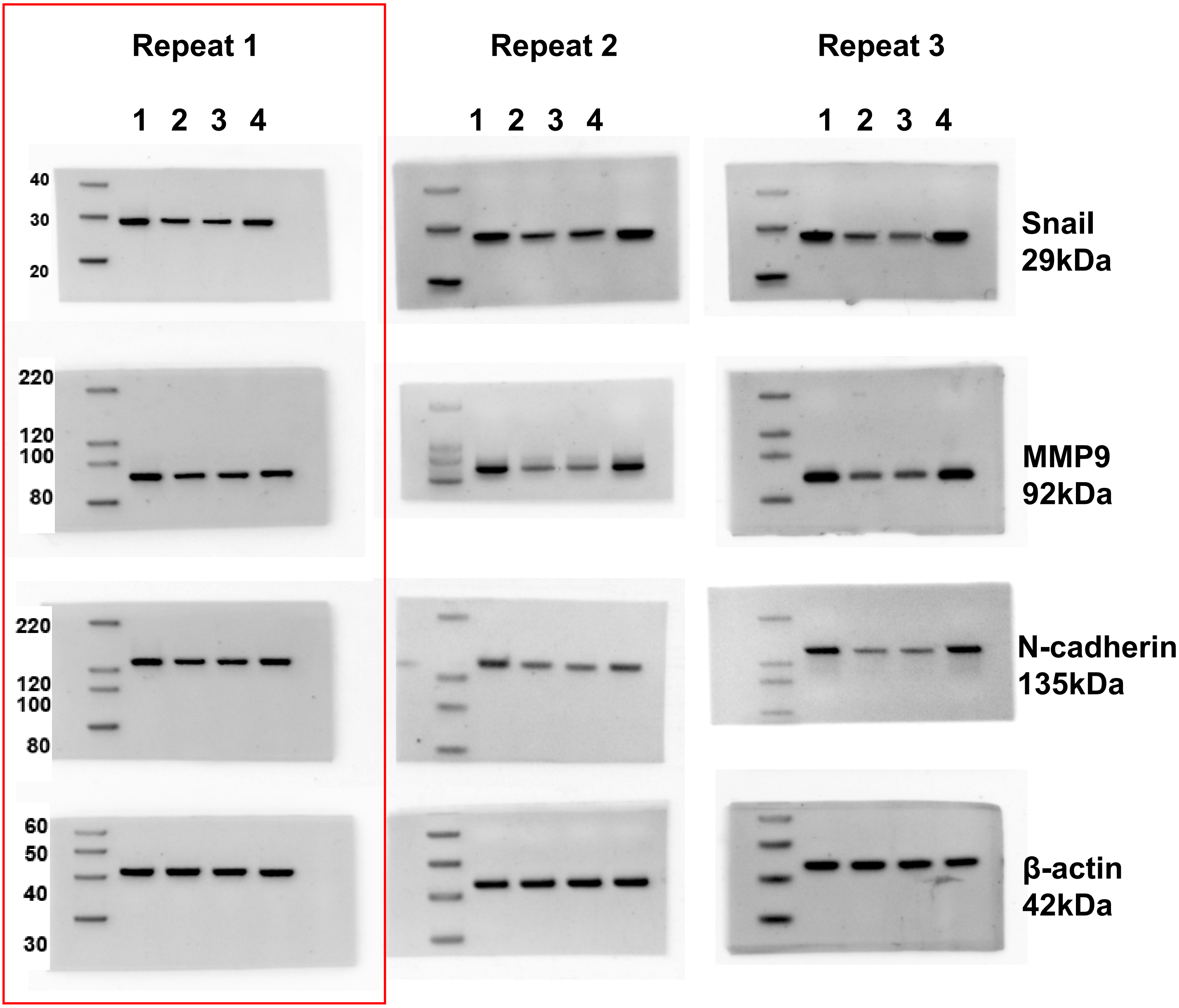

Fig 2I
A2780

1 si-NC
2 si-circ_0001741
3 si-circ_0001741+anti-miR-NC
4 si-circ_0001741+anti-miR-491-5p

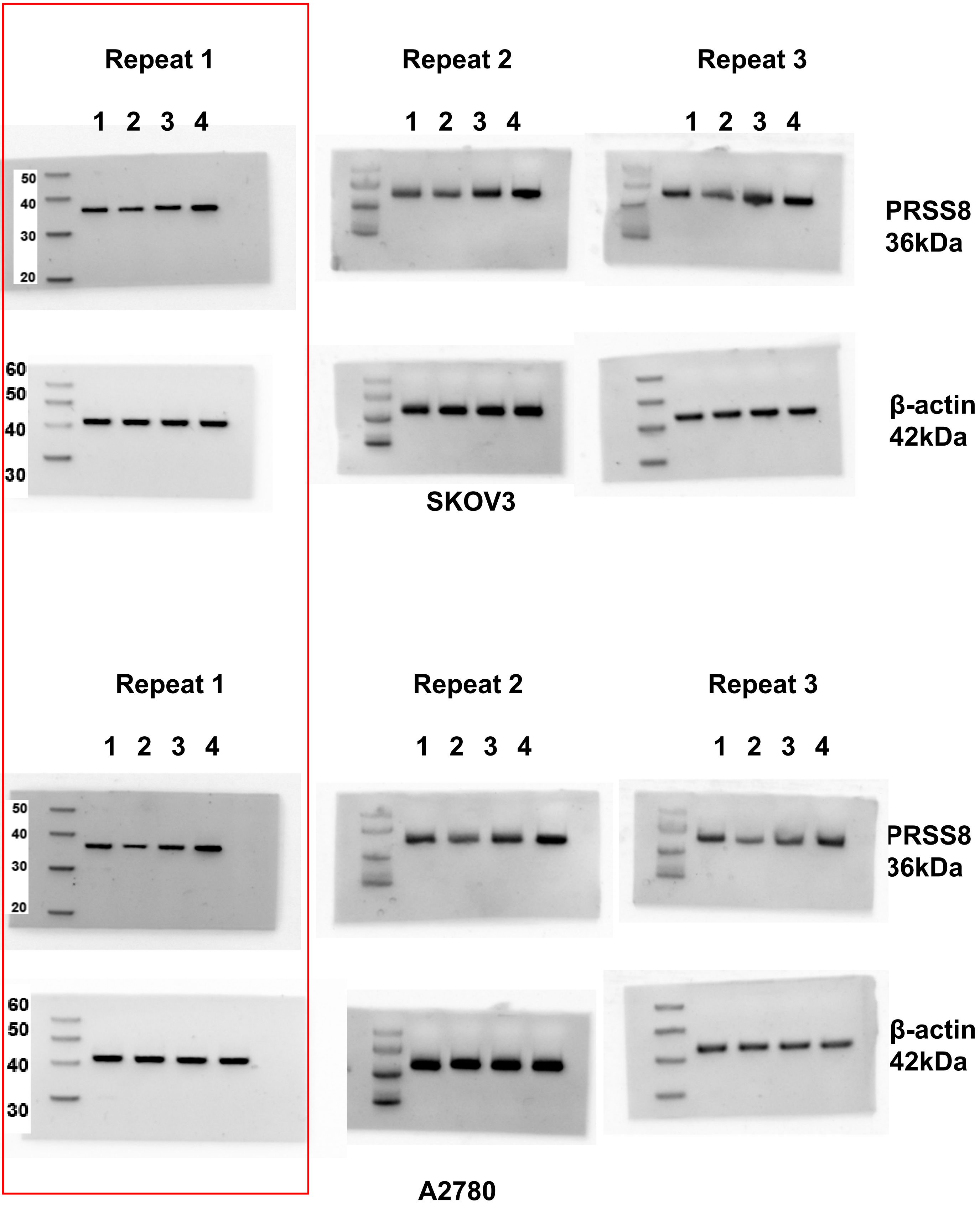

Fig 5G

1 miR-NC
2 miR-491-5p
3 anti-miR-NC
4 anti-miR-491-5p

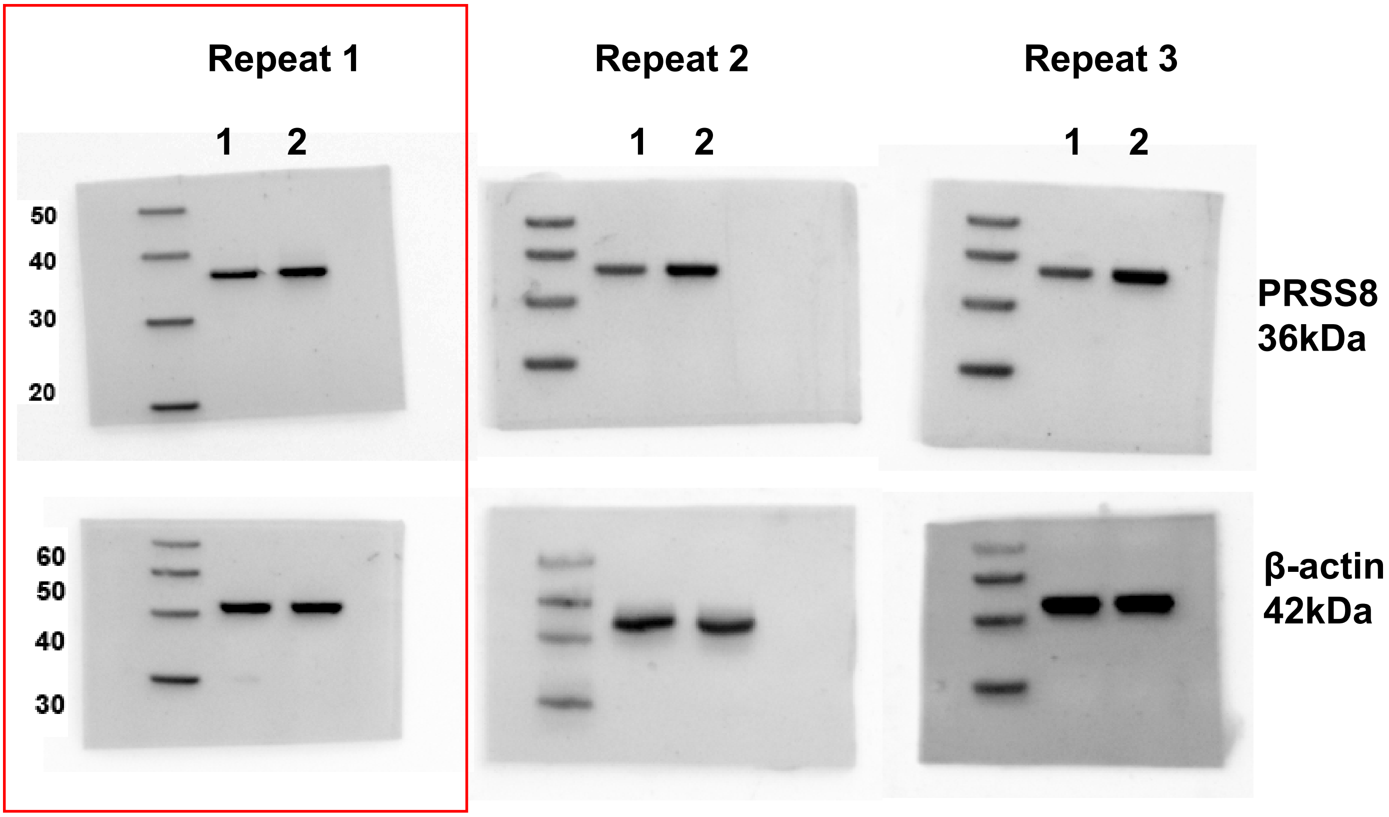

Fig 5I

1 Normal
2 Tumor

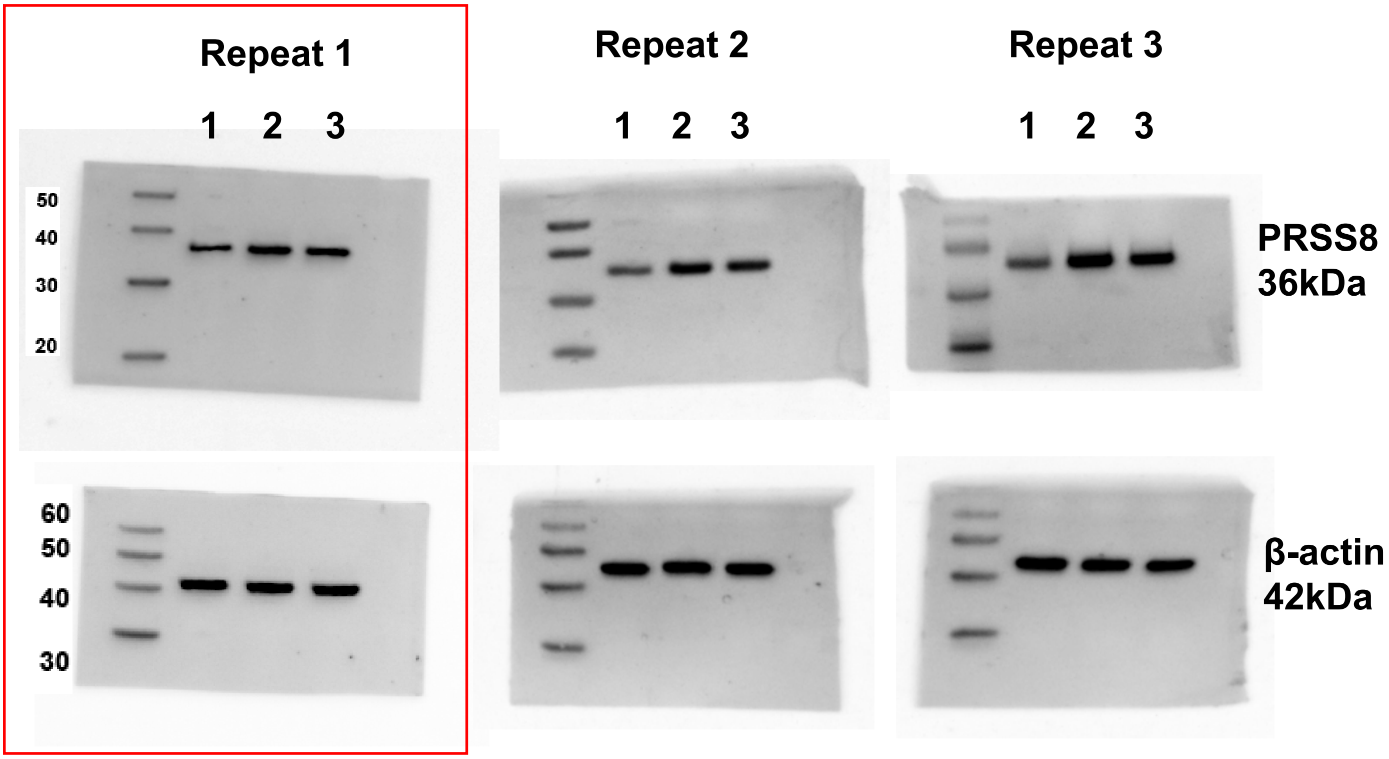

Fig 5K

1 IOSE-80
2 SKOV3
3 A2780

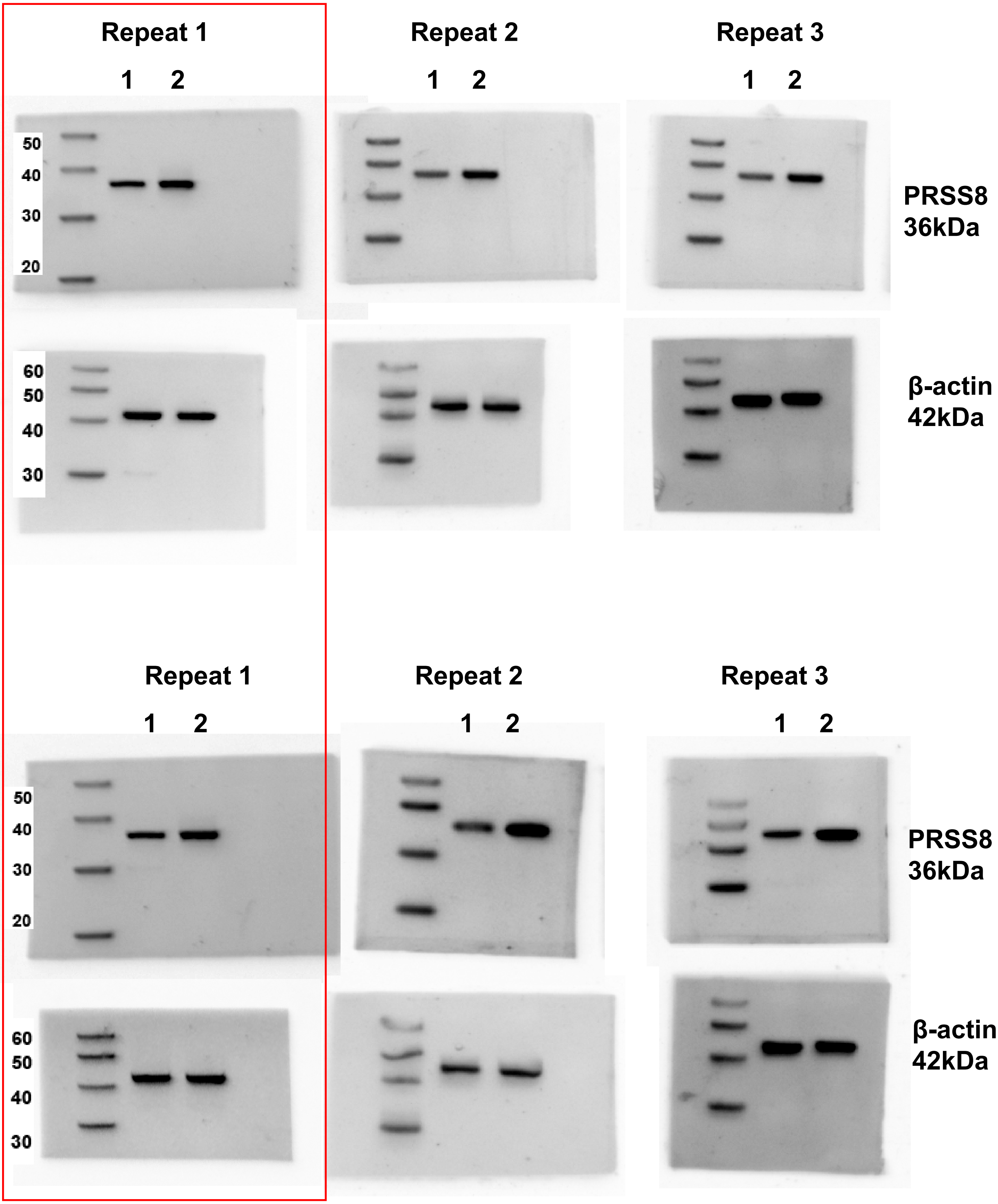

Fig 6B

1 pcDNA
2 pcDNA-PRSS8

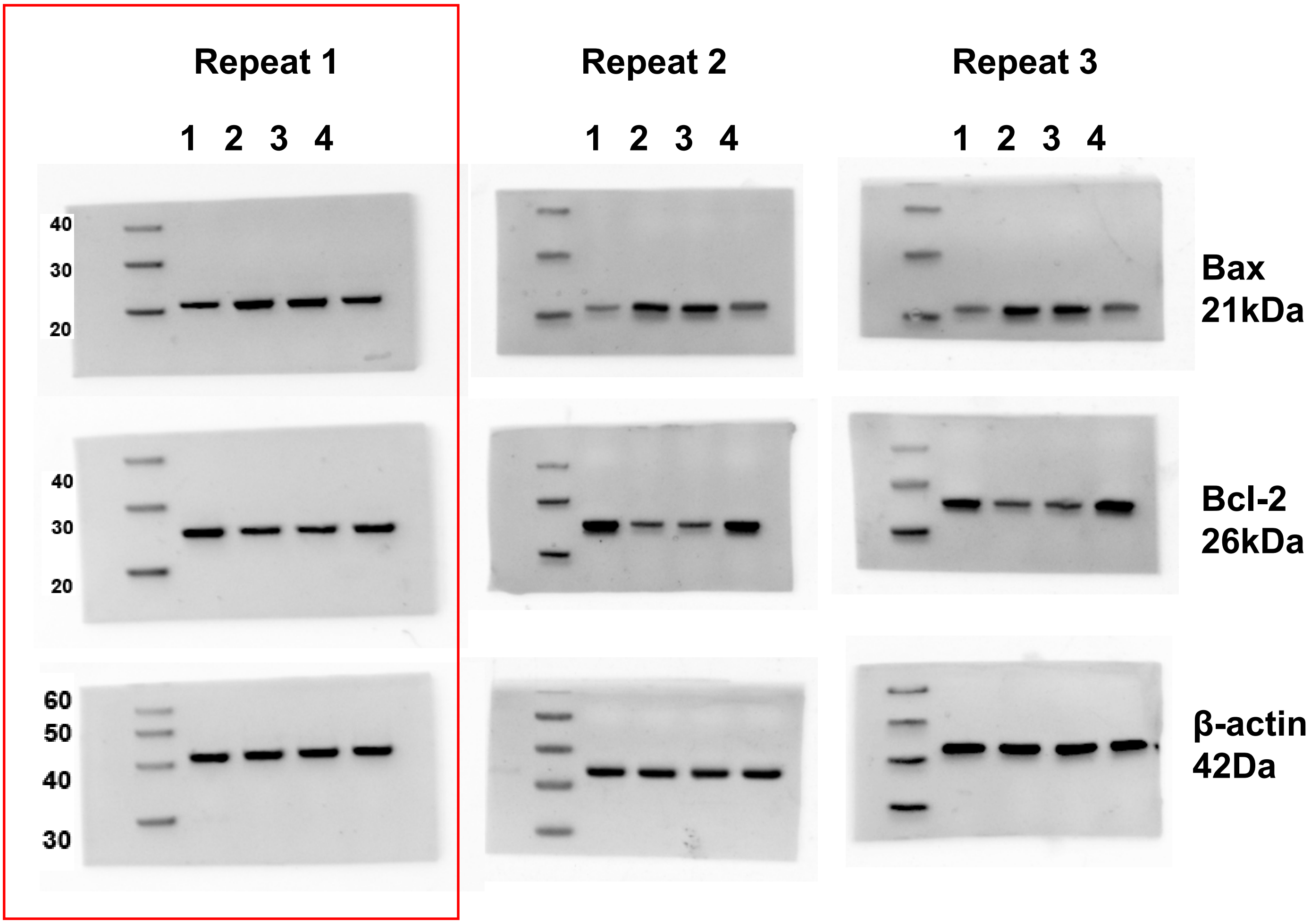

Fig 6G
SKOV3

1 miR-NC
2 miR-491-5p
3 miR-491-5p+pcDNA
4 miR-491-5p+pcDNA-PRSS8

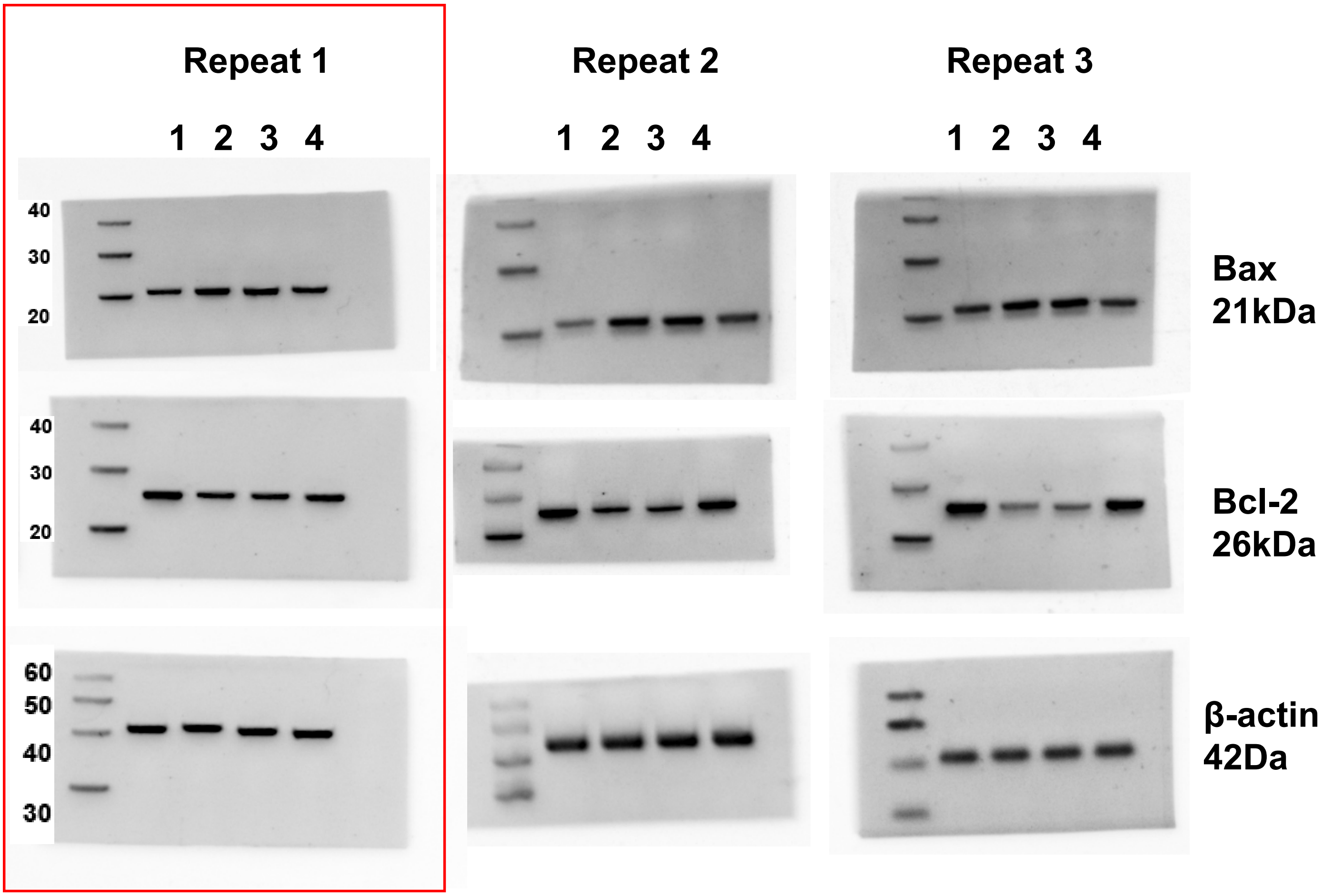

Fig 5G
A2780

1 miR-NC
2 miR-491-5p
3 miR-491-5p+pcDNA
4 miR-491-5p+pcDNA-PRSS8

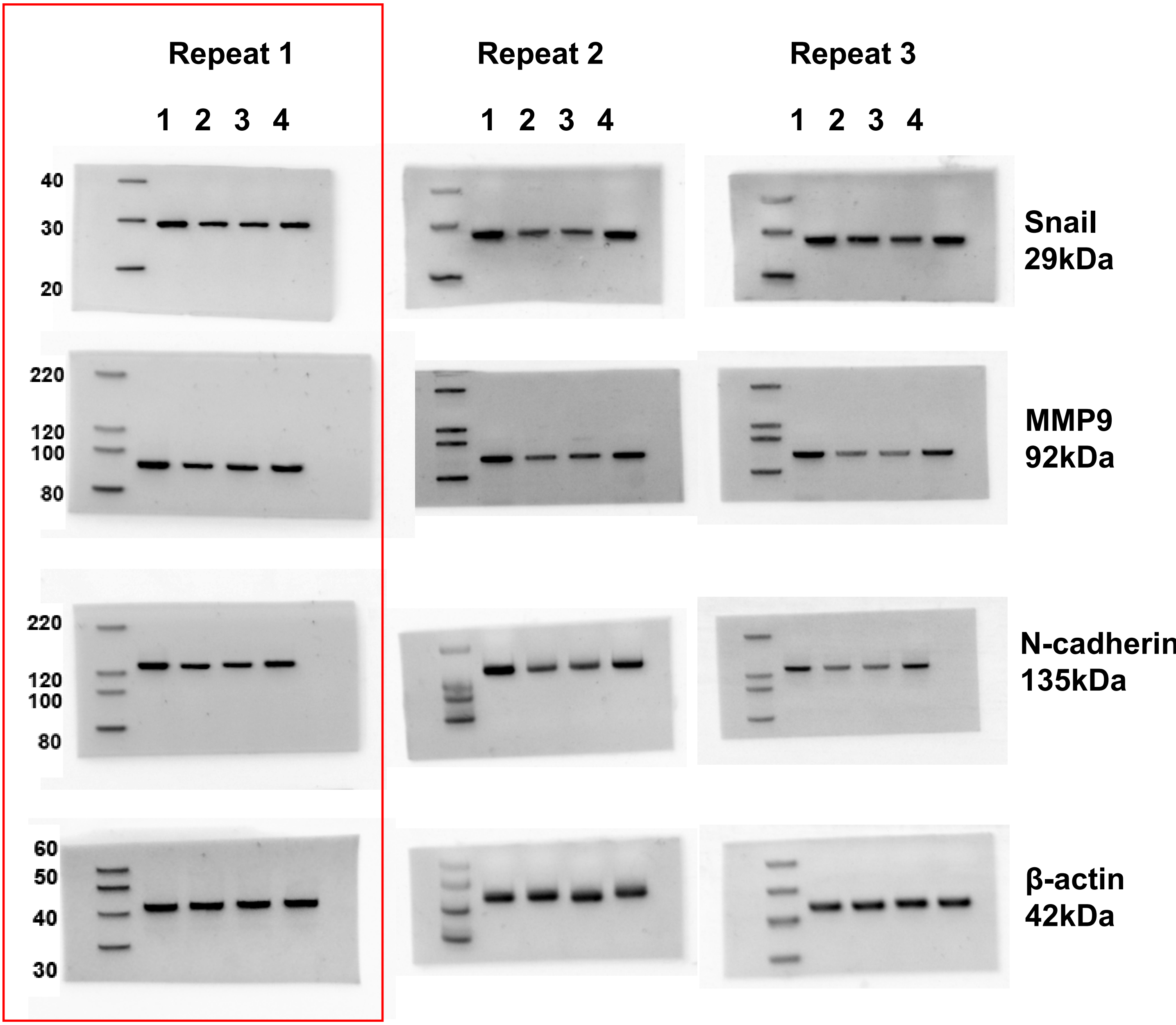

Fig 5J
SKOV3

1 miR-NC
2 miR-491-5p
3 miR-491-5p+pcDNA
4 miR-491-5p+pcDNA-PRSS8

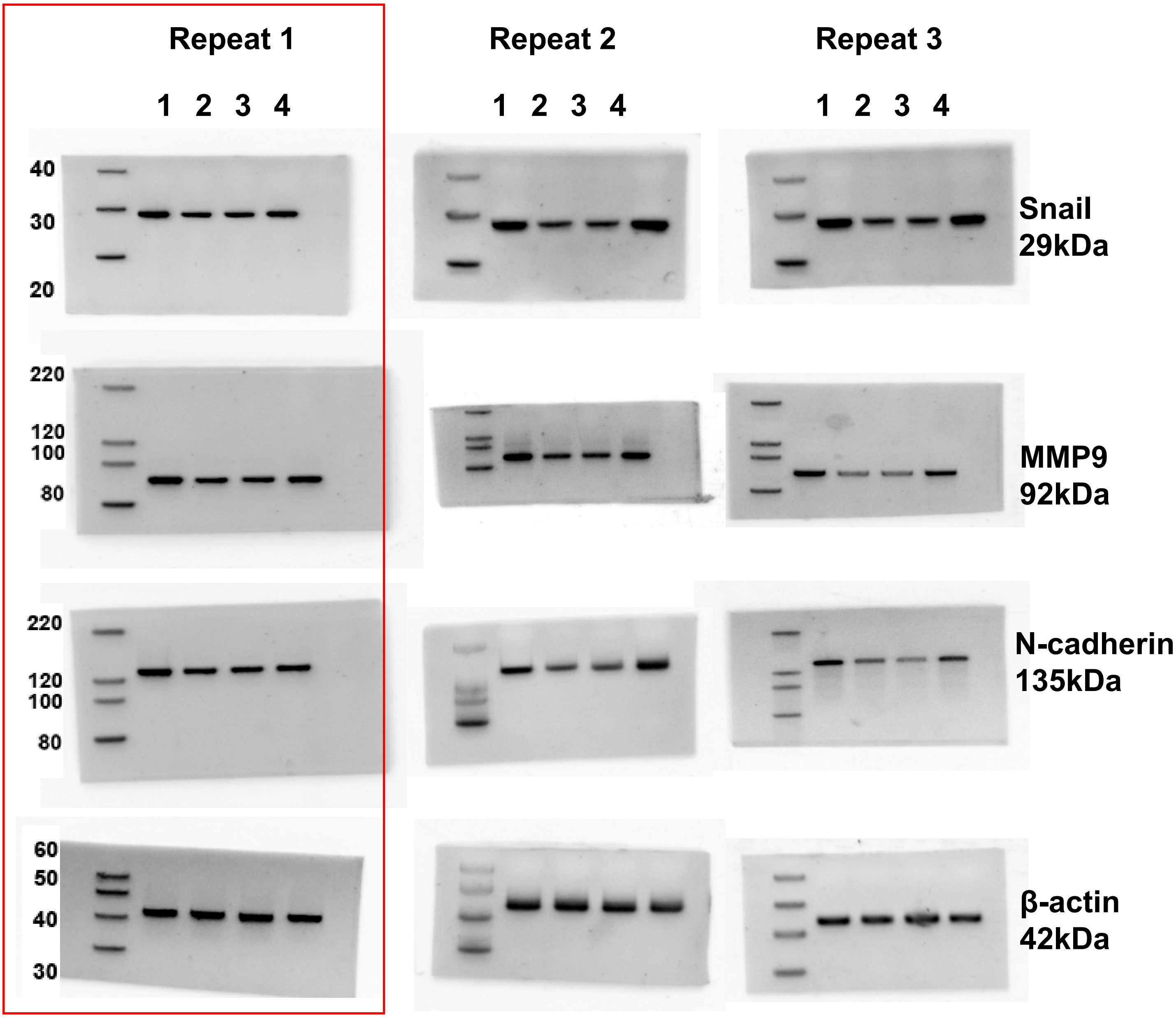

Fig 5J
A2780

1 miR-NC
2 miR-491-5p
3 miR-491-5p+pcDNA
4 miR-491-5p+pcDNA-PRSS8

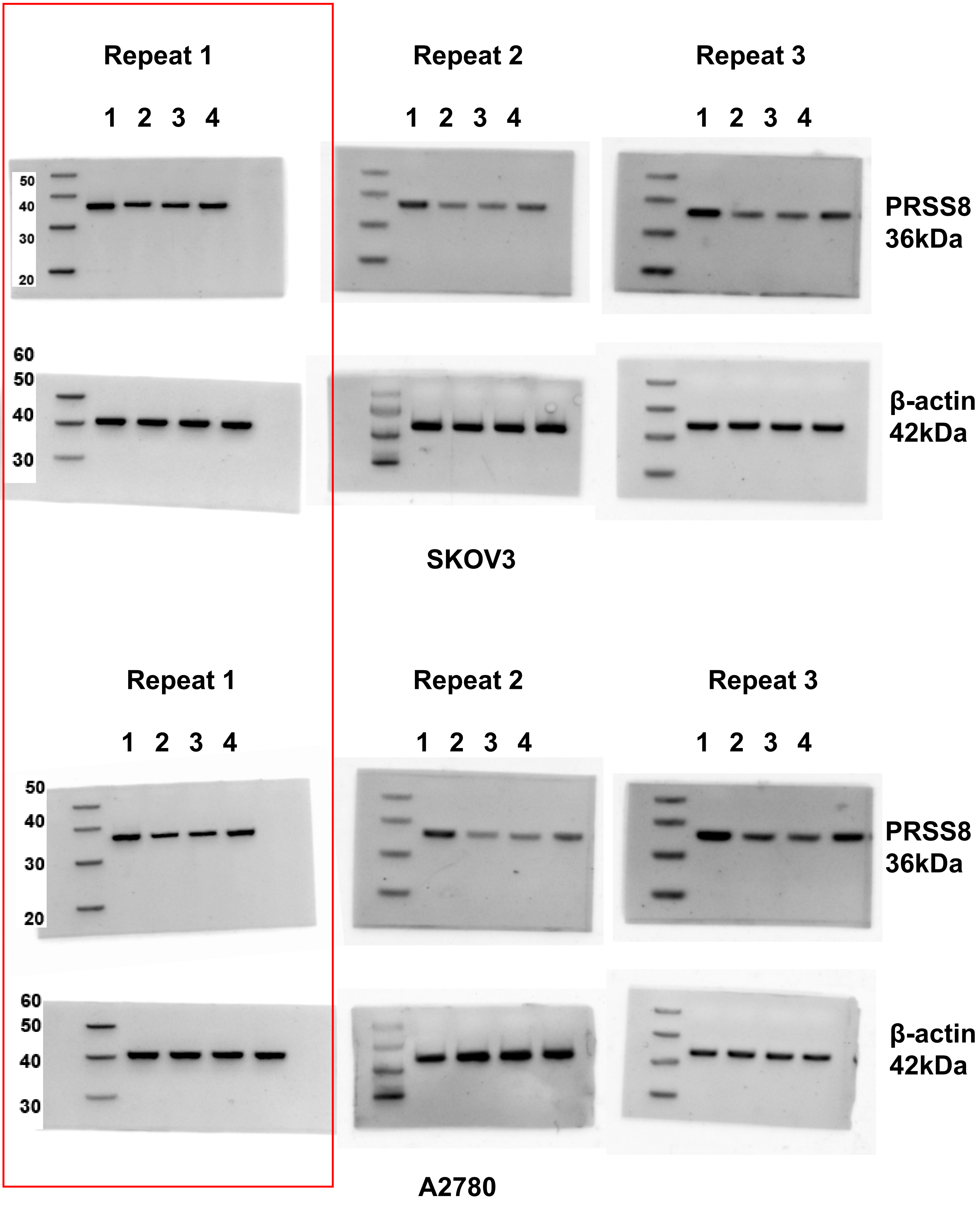

Fig 7B

1 si-NC
2 si-circ_0001741
3 si-circ_0001741+anti-miR-NC
4 si-circ_0001741+anti-miR-491-5p

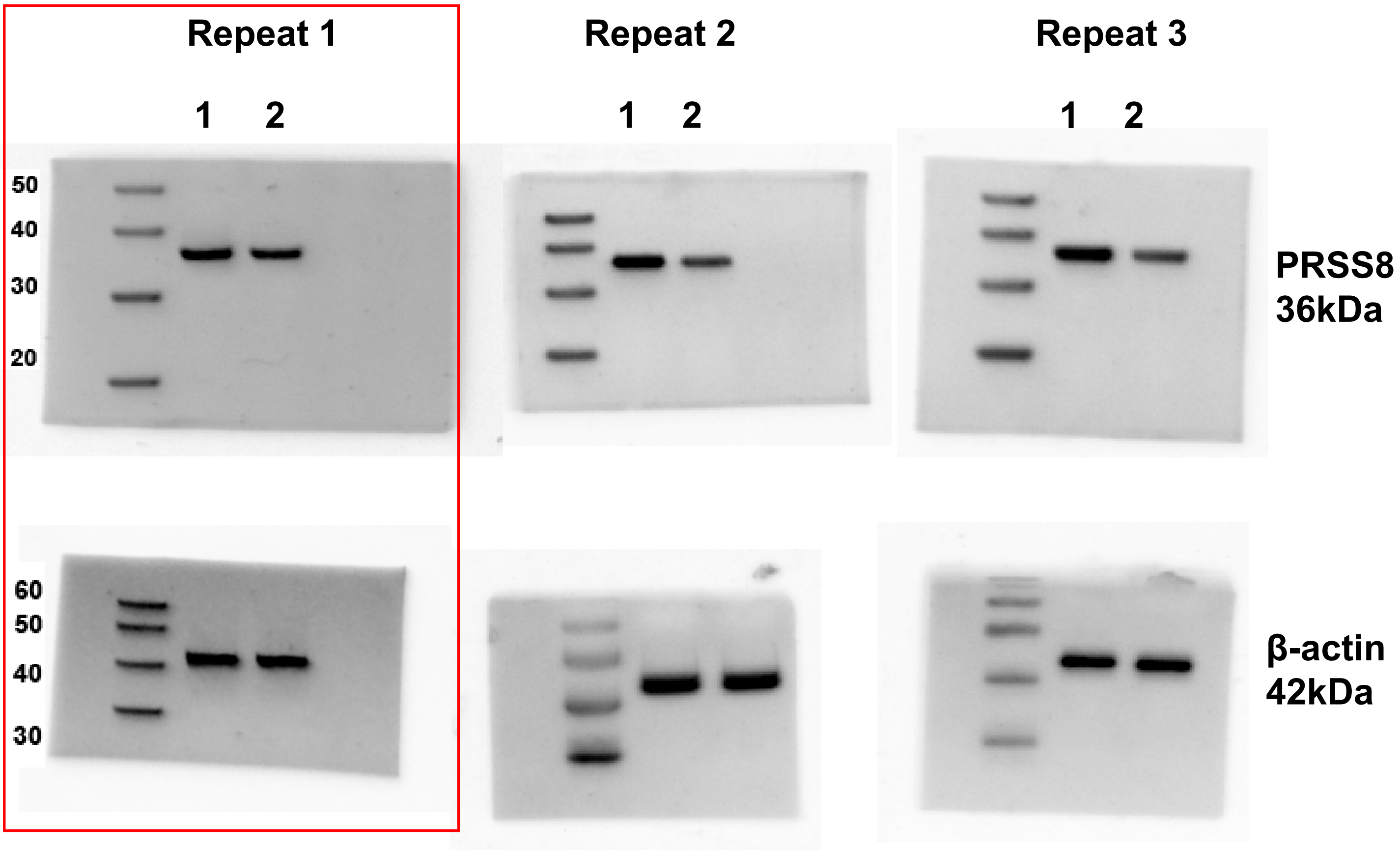

Fig 8F

1 sh-NC
2 sh-circ_0001741
