# Supplementary figures and images for: Circ_0001741 exerts as a tumor promoter in ovarian cancer through the regulation of miR-491-5p/PRSS8 axis
Source: Discov Oncol. 2024 Nov 11;15:643. doi: 10.1007/s12672-024-01474-3 (PMC11554978; doi:10.1007/s12672-024-01474-3)

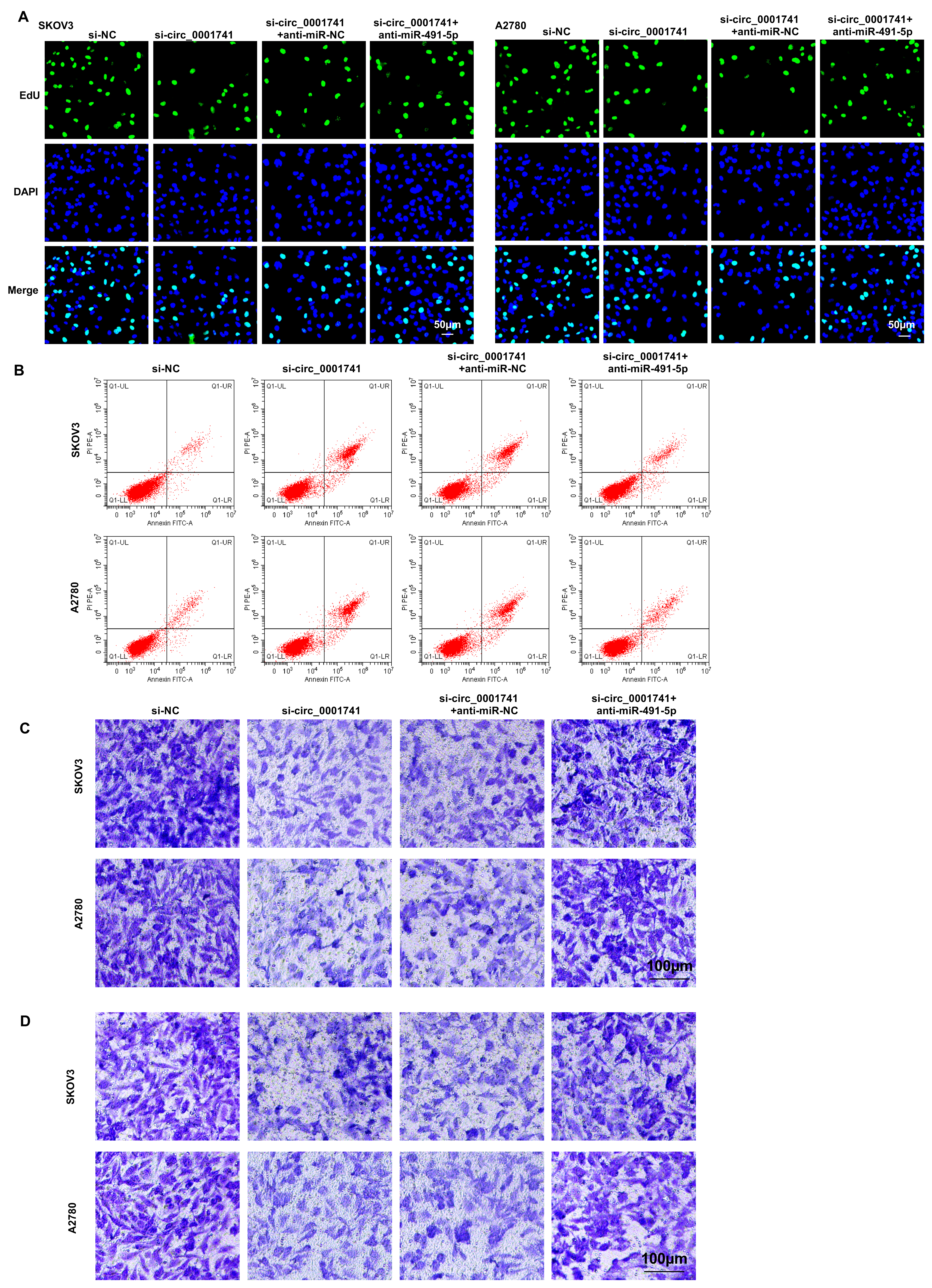

Supplement: Supplementary file 2 — Additional file2 [file 12672_2024_1474_MOESM2_ESM.tif]

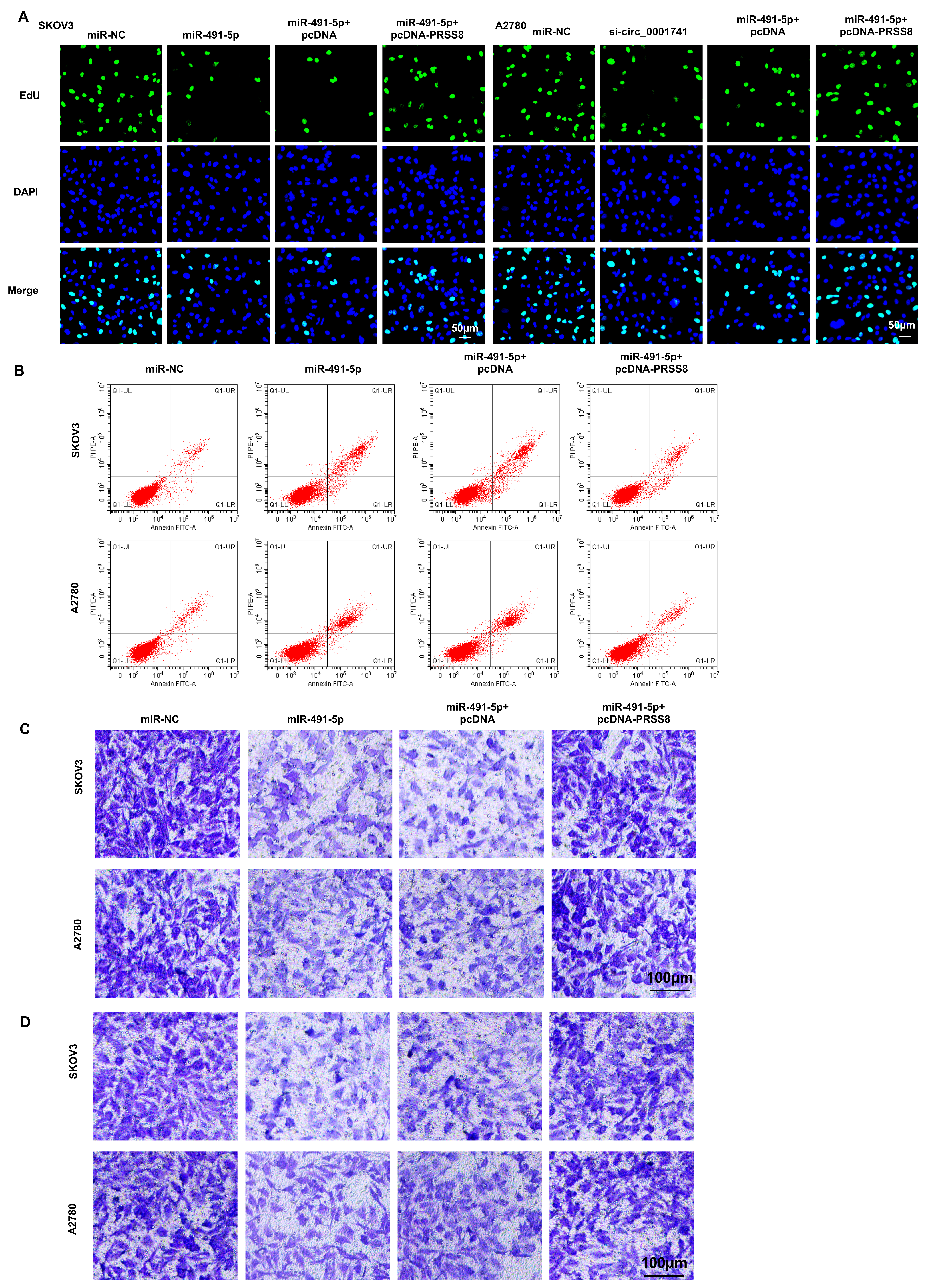

Supplement: Supplementary file 3 — Additional file3 [file 12672_2024_1474_MOESM3_ESM.tif]
